# Proton pump inhibitor exposure modulates functional and transcriptional responses in *Lactobacillus acidophilus*: a comprehensive computational and experimental insights

**DOI:** 10.3389/fcimb.2026.1781831

**Published:** 2026-05-08

**Authors:** Romen Singh Naorem, Kalpajit Dutta, Sudipta Sankar Bora, Anju Barhai Teli

**Affiliations:** 1 Multidisciplinary Research Unit, Jorhat Medical College and Hospital, Jorhat, India; 2 Department of Biochemistry, Jorhat Medical College and Hospital, Jorhat, India

**Keywords:** drug targets, *Lactobacillus acidophilus*, molecular dynamics, off-targets, proton pump inhibitors, transcriptional repression

## Abstract

Proton pump inhibitors (PPIs) are among the most widely prescribed medications for gastric acid-related disorders. However, their effect on the gut microbiota remains incompletely understood, despite emerging evidence suggesting potential long-term alterations in microbial composition and reductions in beneficial taxa. In this study, *Lactobacillus acidophilus*, a well-known probiotic species, was used as a representative model organism to investigate the microbiological effects of PPIs. This specific bacterium is linked to immune modulation, vitamin metabolism, and the preservation of the epithelial barrier. The effects of PPIs on *L. acidophilus* at the structural and functional levels were elucidated by an integrated framework including subtractive genomics, molecular docking, molecular dynamics (MD) simulations, antimicrobial assays, and transcriptional analysis. Using a multi-criteria scoring system, essential, non-redundant, non-human homologous cytoplasmic proteins were ranked and mapped to important pathways such as ATP synthesis, peptidoglycan biosynthesis, amino-sugar metabolism, nucleotide metabolism, and protein maturation. Molecular docking suggested potential binding of pantoprazole and rabeprazole to targets such as MurA, MurB, MurE, GlmS, NadE, AtpD, Def, and PyrH proteins. MD simulation showed stable protein-PPI complexes with localized flexibility changes near catalytic domains while preserving the global fold. Consistent with *in-silico* expectations, both pantoprazole and rabeprazole exhibited dose-dependent growth inhibition of *L. acidophilus*, whereas qRT-PCR revealed transcriptional downregulation of genes involved in cell-wall production, NADH metabolism, and energy generation. Pantoprazole elicited the most uniform transcriptional suppression, whereas rabeprazole had stronger but more varied effects. The present findings provide preliminary insights into potential interactions between PPIs and probiotic bacteria at the molecular and cellular levels. However, the results reflect species-specific responses under *in vitro* conditions and should be interpreted cautiously, as transcriptional changes do not directly confirm functional inhibition and the concentrations tested may represent upper-range exposure scenarios. Further *in vivo* and multi-species studies are required to validate this observation and better understand their clinical implications for microbiome stability during PPI therapy.

## 1 Introduction

The human gastrointestinal tract hosts a vastly diverse and continuously evolving community of microbes, commonly known as the gut microbiota. This microbial community covers bacteria, archaea, fungi, protozoa, and viruses, collectively accounting for an estimated 10¹³-10¹^4^ cells and more than three million different genes (Minagar and Jabbour, 2025). Gut microbial colonization begins at birth and progressively alters throughout life in response to environmental, nutritional, physiological, and lifestyle influences (Parizadeh and Arrieta, 2023). The maintenance of a balanced gut microbiota is critical for gastrointestinal integrity and whole-body health. The indigenous gut microbiota supports host physiology by fermenting otherwise indigestible nutrients, producing vital vitamins, influencing bile acid pathways, and modulating immune activity (Valencia et al., 2025). In addition, the gut microbiota strengthens epithelial barrier integrity and limits pathogen colonization by outcompeting harmful microorganisms. Alterations in gut microbial equilibrium, often defined as dysbiosis, have been implicated in a wide range of ailments, including metabolic dysfunction, inflammatory bowel disease, neurodegenerative disorders, allergy diseases, and susceptibility to infections (Di Vincenzo et al., 2024).

Within the diverse community of health-promoting gut microbes, probiotics have emerged as a prominent focus of scientific investigation. Probiotics refer to viable microorganisms that exert beneficial effects on host health when administered in appropriate amounts (Maftei et al., 2024). Members of genera such as *Lactobacillus, Bifidobacterium, Saccharomyces*, and *Streptococcus* are generally known for enhancing barrier integrity, modulating immune activity, producing antimicrobial metabolites, and improving microbial imbalances associated with dysbiosis (Fijan, 2014). Accordingly, probiotics are widely employed as complementary therapies for gastrointestinal diseases, antibiotic-related diarrhoea, metabolic abnormalities, and immunological instability (Sarita et al., 2025).

Gut microbial composition and function are extremely susceptible to internal and external variables, including mode of delivery at birth, nutrition, age, stress, geography, host genetics, antibiotic exposure, and lifestyle practices such as tobacco and alcohol consumption (Khalil et al., 2024). Recent findings further reveal that drugs created for non-microbial targets may nonetheless exert large effects on gut microbiota architecture (Grießhammer et al., 2025). Emerging evidence indicates that regularly used pharmaceuticals can modulate microbial physiology, metabolism, and gene expression, highlighting the importance of drug-microbe dynamic interactions within the gut ecosystem (Tian et al., 2023). Notably, proton pump inhibitors, which are commonly used to treat peptic ulcer disease and gastroesophageal reflux disease (GERD), have been shown to significantly disturb the gut microbiome due to their strong suppression of stomach acidity (Jackson et al., 2016; Fossmark and Olaisen, 2024).

PPIs are among the most widely prescribed drugs globally and are indicated for conditions such as GERD, Zollinger–Ellison syndrome, peptic ulcer disease, and other acid-related disorders (Scarpignato et al., 2016; Katz et al., 2022). These drugs exert their therapeutic effects by irreversibly blocking the stomach H^+^/K^+^-ATPase in parietal cells, causing persistent inhibition of gastric acid output and protection against acid-induced mucosal injury (Mullin et al., 2009; Shin and Kim, 2013; Freedberg et al., 2014; Scarpignato et al., 2016). However, rising concerns have surfaced regarding the influence of long-term PPI medication on gut microbiota homeostasis, including modifications in probiotic populations (Macke et al., 2020). These compounds potentially alter gut microbiota composition and function, contributing to dysbiosis and increased susceptibility to gastrointestinal disorders (Tasnim et al., 2020; Tian et al., 2023).

Multiple studies have revealed increased luminal pH, reduced microbial diversity, proliferation of potentially pathogenic species, and declined beneficial bacteria abundance in PPI users. These microbiota disruptions have been associated with increased risks of *Clostridioides difficile* infection, small intestine bacterial overgrowth (SIBO), metabolic disturbances, and compromised immunological responses (Chen et al., 2016; Imhann et al., 2016; Jackson et al., 2016; Elghannam et al., 2024). Such findings highlight increasing apprehensions about the off-target effects of PPIs on commensal microbial communities vital for intestinal homeostasis.

*Lactobacillus acidophilus* is a well-known probiotic microorganism that inhabits the human gastrointestinal tract (GIT) and is a significant component of the gut microbiota. This bacterium is prominent for its role in maintaining epithelial barrier integrity, modulating immune responses, and contributing to metabolic homeostasis through the production of beneficial metabolites such as short-chain fatty acids and vitamins (Heeney et al., 2018; Al-Sadi et al., 2021; Dempsey and Corr, 2022). Several studies have reported that the abundance of *Lactobacillus* species is altered in individuals undergoing long-term PPI therapy, suggesting that these drugs may influence beneficial gut microbial populations (Vesper et al., 2009; Freedberg et al., 2014; Jackson et al., 2016; Hojo et al., 2018; Kiecka and Szczepanik, 2023). Despite its recognized probiotic value, the molecular mechanisms via which PPIs influence *L. acidophilus* physiology and gene regulation remain poorly understood, particularly with respect to direct interactions with crucial bacterial proteins. Based on its biological relevance and well-established probiotic functions, *L. acidophilus* was selected in this study as a representative model organism to investigate potential PPI-microbe interactions.

Although PPIs are designed to exclusively inhibit the gastric proton pump, emerging evidence indicates that they may interact with bacterial proteins possessing analogous or functionally relevant domains (Blandón-Arias et al., 2025; Cao et al., 2025; Talukdar et al., 2025). Systematic evaluation of such pharmacological off-target interactions is therefore warranted. Computational approaches, including molecular docking and molecular dynamics simulations, provide effective tools for predicting drug-protein interactions and identifying bacterial pathways susceptible to pharmacological interference (Santos et al., 2019). Proteins involved in cell wall biosynthesis, nucleotide metabolism, energy production, and vitamin or cofactor synthesis are critical for bacterial survival and adaptation, and unintentional targeting of these pathways could compromise *L. acidophilus* viability and probiotic function (Gerdes et al., 2002; Zhang and Lin, 2009; Typas et al., 2012; Mobegi et al., 2014; Goncheva et al., 2022). PPIs are prodrugs that generally need protonation (acidic activation) to generate reactive intermediates capable of inhibiting the gastric H^+^/K^+^-ATPase (Shin and Sachs, 2008). Such activation is limited in near-neutral conditions, and PPIs may exist in altered or less active forms. Nevertheless, recent studies have reported that these substances, as well as their intermediate or degradation forms, may also interact non-canonically with biological molecules or systems outside of the gastric environment (Wołowiec et al., 2025).

This study applied an integrative computational and experimental approach to analyse the structural and functional effects of PPI exposure on gut microbiota-relevant bacteria, using *L. acidophilus* as a representative model organism. A subtractive genomics technique discovered essential, conserved proteins as potential interaction targets. Molecular docking and dynamics simulations with therapeutically relevant PPIs (omeprazole, pantoprazole, rabeprazole, and lansoprazole) evaluated binding affinities and complex stability. Computational predictions were reinforced by targeted gene expression investigations to determine how such interactions influence bacterial physiology, particularly in pathways related to cell wall integrity, nucleotide and energy metabolism, vitamin biosynthesis, and stress responses. By integrating pharmacological insights with microbial systems biology, the study provides mechanistic understanding of PPI-induced perturbations in *L. acidophilus* and advances knowledge of drug-microbe interactions with potential clinical implications. Emphasis was placed on direct molecular and transcriptional effects on essential bacterial pathways, rather than a comprehensive physiological or ecological assessment, through the combination of *in-silico* predictions and targeted gene expression validation of key enzymes.

## 2 Materials and methods

### 2.1 Extraction of complete genomic datasets for *L. acidophilus*

**Figure 1:**
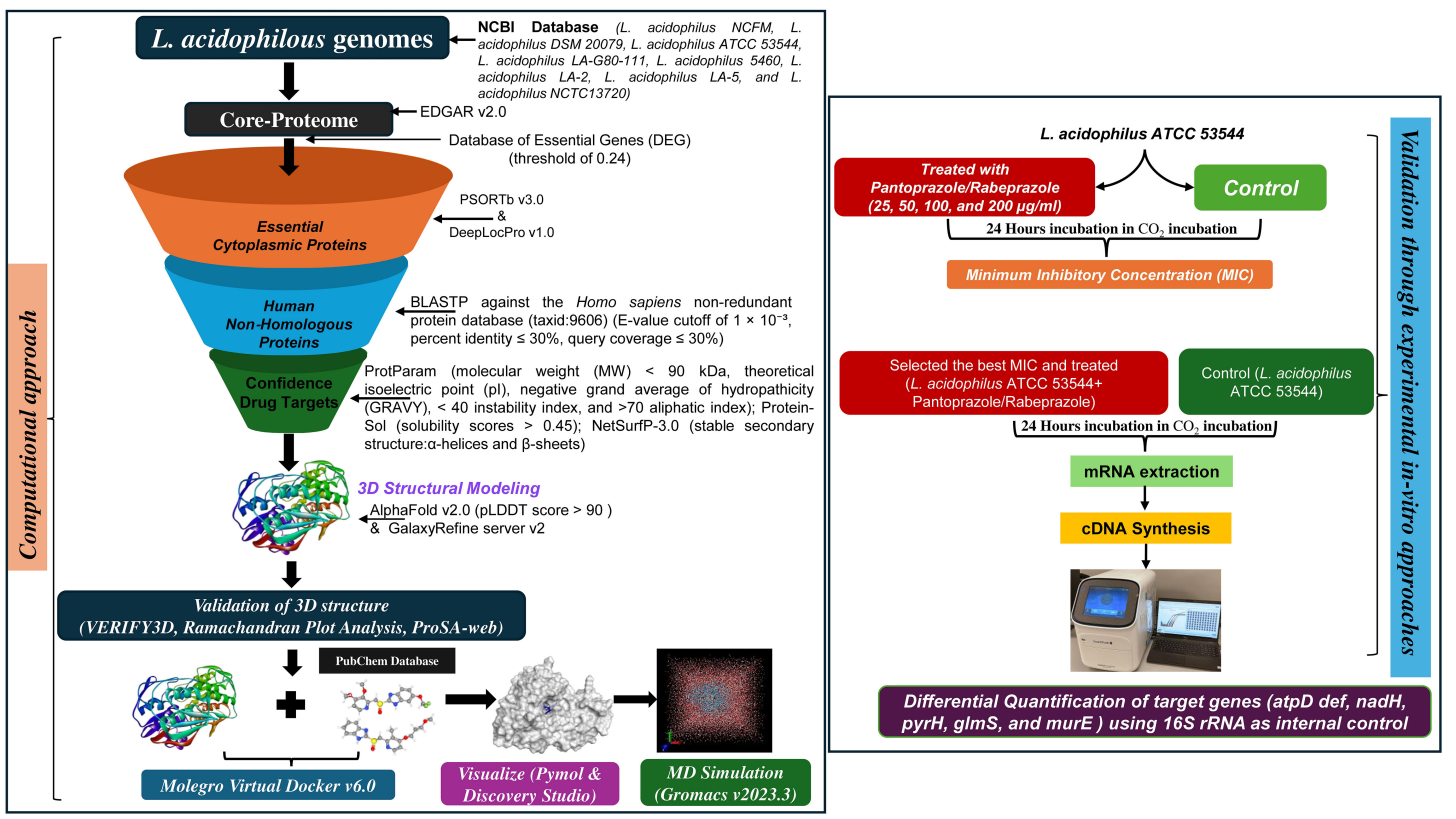
Infographic showing a computational approach to identify drug targets in *L. acidophilus* genomes, including genome analysis, identification of essential and non-homologous proteins, 3D structural modeling, molecular docking and dynamics. Experimental validation involves treating *L. acidophilus* ATCC 53544 with pantoprazole/rabeprazole, determining minimum inhibitory concentration, extracting mRNA, synthesizing cDNA, and quantifying target gene expression using 16S rRNA as control.

The overall workflow adopted in this study, integrating both computational target identification and experimental validation in *Lactobacillus acidophilus*, is illustrated in **Figure 1** and is described in detail in the following sections. The complete genome sequences of *L. acidophilus* strains were retrieved from the NCBI GenBank database for downstream analysis (https://www.ncbi.nlm.nih.gov/datasets/genome/?taxon=1579). The genomes included *L. acidophilus* NCFM (CP000033), *L. acidophilus* DSM 20079 (CP020620), *L. acidophilus* ATCC 53544 (CP022449), *L. acidophilus* LA-G80-111 (CP054559), *L. acidophilus* 5460 (CP090415), *L. acidophilus* LA-2 (CP102533), *L. acidophilus* LA-5 (CP102518), and *L. acidophilus* NCTC13720 (LR134325).

### 2.2 Prediction of core proteome and removal of redundant proteins

The core proteome of *L. acidophilus* was identified using reciprocal best BLAST hits of all coding sequences (CDS) in EDGAR v3.0 (https://edgar3.computational.bio.uni-giessen.de) (Dieckmann et al., 2021) applying a default E-value threshold of 1 × 10^-5^ and a minimum alignment coverage of 70%, and the core proteome was defined as genes present in all analysed genomes. The core proteome was defined as the set of genes present in all analysed genomes, with *L. acidophilus* ATCC 53544 (CP022449) serving as the reference strain. Paralogs and redundant sequences were removed using the CD-HIT suite (Fu et al., 2012) with a sequence identity threshold of 70%. Representative sequences from each cluster were retained for further analysis.

### 2.3 Identification of essential, cytoplasmic, and human non‐homologous proteins

The non‐redundant core proteome was queried against the Database of Essential Genes (DEG) via GEPTOP 2.0 (Wen et al., 2019) using an essentiality score threshold of 0.24. Subcellular localization of essential proteins was determined using PSORTb v3.0 (https://psort.org/psortb/) (Yu et al., 2010), CELLO2GO (https://cello.life.nctu.edu.tw/cello2go/) (Yu et al., 2014) and DeepLocPro v1.0 (https://services.healthtech.dtu.dk/services/DeepLocPro-1.0/) (Moreno et al., 2024). For PSORTb predictions, a localization score of **≥** 7.5 was considered a high-confidence assignment. CELLO2GO predictions were interpreted based on the highest reliability score for Gene Ontology-supported cellular component annotations, while DeepLocPro predictions with confidence probabilities ≥ 0.5 were considered reliable. Proteins predicted to localize in the cytoplasm by these tools were selected for further analysis, as these are more accessible and essential for bacterial survival (Mondal et al., 2015; Naorem et al., 2022, Naorem et al., 2024). Cytoplasmic protein sequences were screened against the *Homo sapiens* non-redundant protein database (taxid:9606) using BLASTP to identify non-homologous targets. The search was performed with an E-value cutoff of 1 × 10^-^³, percent identity ≤ 30%, and query coverage ≤ 30%, using a word size of 6 and the BLOSUM62 substitution matrix (Lu et al., 2024). Proteins that did not show significant similarity to human proteins based on these criteria were retained for further analysis.

### 2.4 Systematic computational multi-criteria prioritization of confidence drug targets

Physicochemical properties of the candidate proteins were calculated using ProtParam (https://web.expasy.org/protparam/) (Gasteiger et al., 2003), including molecular weight (MW), theoretical isoelectric point (pI), grand average of hydropathicity (GRAVY), instability index, and aliphatic index. Proteins with a molecular weight < 90 kDa were chosen, as lower molecular weight proteins exhibit improved crystallization and structure stability (Solanki and Tiwari, 2018). Proteins with a negative GRAVY value were selected due to their greater hydrophilicity and improved solubility. Protein stability was assessed using the instability index, and proteins with threshold value < 40 were considered (Gamage et al., 2019), while proteins with a high aliphatic index (>70) were preferred because of their potential thermostability (Ikai, 1980). Protein-Sol tool was used to predict solubility; proteins with scores > 0.45 were considered and these proteins are more soluble than the average *Escherichia coli* protein (Hebditch et al., 2017). Proteins surpassing this threshold were prioritized as suitable candidates for heterologous expression and subsequent downstream analyses. DeepTMHMM - 1.0 web tool (https://services.healthtech.dtu.dk/services/DeepTMHMM-1.0/) was used to predict Transmembrane (TM) helices (Hallgren et al., 2022). Proteins without TM domains were prioritized to facilitate downstream purification. Signal peptides and secretory pathways (Sec/SPI) were assessed using SignalP v6.0 (https://services.healthtech.dtu.dk/services/SignalP-6.0/) (Teufel et al., 2022) with cutoff value of zero, since secreted proteins often play key roles in pathogenesis and can serve as druggable targets. Additionally, protein structural features were evaluated with NetSurfP-3.0 (https://services.healthtech.dtu.dk/services/NetSurfP-3.0/), which predicts solvent accessibility, secondary structure, intrinsic disorder, and backbone dihedral angles (φ/ψ). Proteins with ≥30% solvent-accessible residues, limited long disordered regions (<20 consecutive residues), stable secondary structure (α-helices and β-sheets), and minimal disordered regions were considered favourable for structural modelling (Høie et al., 2022).

To integrate these criteria into a unified prioritization framework, a multi-criteria scoring system was implemented in R (v4.5.2) using the dplyr package (RStudio Team, 2025). Each parameter was assigned equal weight, and a binary scoring scheme was applied: proteins meeting a defined threshold received a score of 1, whereas those failing to meet the threshold received 0. The evaluated parameters included molecular weight, GRAVY, instability index, aliphatic index, solubility score, absence of transmembrane helices, presence of signal peptide features, and favorable structural properties predicted by NetSurfP-3.0. An overall Total Score was calculated as the sum of all individual parameter scores. Proteins achieving a Total Score ≥ 8 were classified as high-confidence druggable targets, while proteins with lower scores were excluded from further prioritization. Because all parameters were assigned equal weights, the ranking reflects a consensus of physicochemical, structural, and solubility characteristics rather than reliance on any single criterion. The robustness of the ranking was evaluated by confirming that top-ranked proteins consistently satisfied the majority of independent filtering criteria within the pipeline.

Lastly, the qualified confidence target proteins were mapped to KEGG Orthology (KO) IDs using KEGG Mapper (Kanehisa and Goto, 2000). This method aided functional annotation by incorporating the proteins into well-characterized biological pathways. Particular emphasis was placed on proteins involved with cell wall biosynthesis, nucleotide biosynthesis, energy metabolism, protein synthesis, and as these processes are universally significant for cellular development and survival (Uddin and Sufian, 2016). Proteins associated with such crucial pathways were prioritized as prospective therapeutic targets, as their inhibition could impair key biological functioning in the bacteria.

### 2.5 Protein-protein interaction network analysis

Proteins involved in cell wall biosynthesis, nucleotide biosynthesis, protein synthesis, and energy metabolism were employed for protein-protein interaction network analysis by STRING v12.0 (confidence score ≥ 0.70) (STRING Consortium, n.d.). The network topology was analysed using the output data of STRING in Cytoscape v3.10.0 to identify hub and regulatory proteins. Functional enrichment analysis, including GO annotations and KEGG pathway mapping, was executed to elucidate the biological roles and pathway associations of the network proteins, facilitating the identification of key proteins involved in core cellular processes. Hub proteins with high centrality, singleton proteins with crucial metabolic functions, and proteins linked to important pathways like cell wall biosynthesis, nucleotide metabolism, energy production, and protein synthesis were chosen from the protein-protein interaction network for downstream analyses in order to capture both central regulators and distinct metabolic effectors.

### 2.6 Three‐dimensional structural modelling

AlphaFold v2.0 was used to create protein 3D structures (Jumper et al., 2021; Cheng et al., 2023). Each model’s quality was evaluated using the predicted Local Distance Difference Test (pLDDT) score, which goes from 0 (low confidence) to 100 (high confidence). According to (Carugo, 2023), residues with pLDDT values less than 50 were regarded as disordered, those with values between 50 and 70 as low confidence, and those with values greater than 70 as trustworthy predictions. Only protein models with an average pLDDT score higher than 90 were subjected to structural refinement using the GalaxyRefine server v2 (https://galaxy.seoklab.org), which enhances stereochemical quality and side-chain conformations, in order to guarantee the selection of robust and well-folded structures. ERRAT (Error Analysis of Non-bonded Interactions), VERIFY3D (Structure Evaluation by 3D-1D Profile Compatibility), PROCHECK (Ramachandran Plot Analysis), and ProSA-web (Protein Structure Analysis) were used to evaluate the revised 3D structures. ERRAT assesses non-bonded interactions; values more than 95 signify high-quality models, while scores over 50 are acceptable (Colovos and Yeates, 1993). VERIFY3D assesses sequence-structure compatibility, with reliable models having ≥80% of residues scoring ≥0.2, although ≥70% is considered acceptable (Lüthy et al., 1992). Ramachandran plots generated by PROCHECK evaluate stereochemical quality, where >90% residues in favoured regions indicate high-quality geometry (Laskowski et al., 2018). ProSA-web (https://prosa.services.came.sbg.ac.at/prosa.php) calculates a Z-score to estimate overall folding quality, with values within -4 to -14 typically corresponding to native-like proteins (Wiederstein and Sippl, 2007). These validations confirmed the reliability of the models for subsequent docking and molecular dynamics simulations.

### 2.7 Molecular docking of prioritized protein targets

The 3D structures of the prioritized protein targets were prepared by rectifying structural inconsistencies including missing atoms, incorrect bond assignments, and improper protonation states in Molegro Virtual Docker (MVD) v6.0. Polar hydrogens were added, and the structures were energy‐minimized to ensure optimal conformations (Bitencourt-Ferreira and de Azevedo, 2019).

The 3D structures of PPI ligands *viz*., pantoprazole (4679), rabeprazole (5029), omeprazole (4594), lansoprazole (3883), dexlansoprazole (9578005), and esomeprazole (9568614) were obtained from PubChem database (Kim et al., 2023) and subjected to energy minimization prior to docking.

Structural druggability of the modelled protein was assessed using FPocketWeb v1.0.1 (https://durrantlab.pitt.edu/fpocketweb/), a web-based interface of the fpocket algorithm that detects and characterizes putative ligand-binding cavities (Le Guilloux et al., 2009). Pockets with favourable druggability scores, volume, and hydrophobic balance were considered suitable for molecular docking analysis. The predicted binding pockets identified by FPocket were used to define the docking search space for subsequent molecular docking simulations. Cavity identification was performed in the 3D protein structure to identify putative active sites, and docking simulations were run using the MolDock Optimizer search method with default parameters (population size = 50, scaling factor = 0.5, crossover rate = 0.9). The resultant protein-ligand complexes were examined for binding affinity, hydrogen bonding interactions, and hydrophobic contacts to assess the stability and specificity of the interactions. PyMOL v2.5.5 (Schrödinger and DeLano, 2020) and Discovery Studio Visualizer 2020 (BIOVIA Discovery Studio - BIOVIA - Dassault Systèmes®, n.d.) were used for visualization and interaction mapping.

### 2.8 Molecular dynamics simulation

The MD simulation was performed to evaluate the dynamic behaviour of protein-ligand complexes and to validate the stability of docking-derived binding poses using GROMACS 2022 (Abraham et al., 2015) with the AMBER99SB force field. This force field was used to accurately model bonded and non-bonded atomic interactions in proteins during molecular dynamics simulations. Acpype server was used to generate compatible ligand topologies (Kagami et al., 2023). Each complex was solvated in a cubic periodic box with the TIP3P explicit water model to provide an explicit solvent environment that mimics the aqueous conditions surrounding biomolecules, maintaining a minimum distance of 1.0 nm between the solute and the box boundary for ensuring sufficient solvent around the protein-ligand complex and prevents artificial interactions between periodic images. System neutrality was achieved by adding counter-ions (Na^+^ or Cl^-^) to maintain electrostatic stability during simulation.

Long-range electrostatic interactions in periodic systems were calculated using the particle mesh Ewald (PME) method with a real-space cut-off of 1.0 nm to balance computation efficiently and accuracy, while Van-der Waals interactions were truncated at 1.0 nm to limit the calculation of short-range dispersion forces while maintaining realistic molecular interactions. All bond lengths were constrained using the Linear Constraint Solver (LINCS) algorithm to allow larger integration time steps and maintain simulation stability. Energy minimization was performed via the steepest descent method until the maximum force was below 1000 kJ/mol/nm to remove steric clashes and unfavourable contracts. Equilibration consisted of a 100 ps NVT ensemble at 300 K using the V-rescale thermostat, followed by a 100 ps NPT ensemble at 1 bar employing the Parrinello-Rahman barostat to control system pressure and adjust the simulation box size. The MD simulations were performed under periodic boundary conditions for a production of 50 nanoseconds (ns) to observe the dynamic behaviour and stability of protein, and protein-ligand complexes. The simulated trajectories were initially analysed in Visual Molecular Dynamics (VMD 2.0) (Humphrey et al., 1996) to ensure proper orientation of the protein-ligand complex within the solvation box and to verify that no artifacts or instabilities occurred during the simulation.

Standard GROMACS tools were used for post-MD simulation analyses. The complexes’ structural stability was evaluated by calculating the root mean square deviation (RMSD) using *gmx rms*, and residue-level flexibility was assessed by calculating the root mean square fluctuation (RMSF) using *gmx rmsf*. Additionally, hydrogen bond contacts were tracked to assess the persistence of important intermolecular interactions with *gmx hbond*, and the radius of gyration (Rg) was calculated using *gmx gyrate* to characterize the overall compactness of the complex throughout the trajectory. XMGrace version 5.1.25 was used to plot and show the data after all dynamic parameters were retrieved from the processed trajectory files (Turner, 2005).

### 2.9 Antimicrobial susceptibility testing

The antimicrobial susceptibility of *L. acidophilus* ATCC 53544 to pantoprazole and rabeprazole was evaluated using the CLSI-recommended broth microdilution method (M45, 3^rd^ edition). The strain was cultured in de Man, Rogosa, and Sharpe (MRS) broth (Oxoid, BD (Becton Dickinson)) with the pH maintained at 5.5 ± 0.2. Cultures were incubated at 37 °C for 24 h under microaerophilic conditions with 5% CO_2_ using a (Galaxy-48R) incubator. An overnight culture was adjusted to 0.5 McFarland and diluted 1:100 in fresh MRS broth to obtain an inoculum of approximately 5 × 10^5^ to 1 × 10^6^ CFU/mL. Twofold serial dilutions of pantoprazole and rabeprazole were prepared in 96-well microtiter plates to achieve final concentrations of 0-200 µg/mL. The concentration range of 0-200 µg/mL was employed to facilitate reliable dose-response analysis. Although intestinal PPI levels after therapeutic dosing are likely lower due to rapid absorption and metabolism (Sachs et al., 2006; Shin and Kim, 2013), transient luminal concentrations may occur following oral administration. Therefore, this range was applied to examine potential drug–microbe interactions under controlled *in vitro* conditions. To minimize degradation, all PPI stock solutions were freshly prepared prior to each experiment and protected from long exposure to light and ambient conditions. PPIs are known to be pH dependent. As such, experimental exposure times were restricted to predetermined incubation times. It ensured the presence of biologically active substances during the evaluation of bacterial growth. Each well contained 100 µL of drug dilution and 100 µL of bacterial inoculum (final volume: 200 µL). Plates were incubated at 37 °C for 48 h under microaerophilic conditions. Appropriate controls were included: media blank (MRS only), and growth control (bacteria without drug). All assays were performed in triplicate. Bacterial growth was quantified by measuring optical density at 600 nm (OD_600_) using a microplate reader. Percentage growth inhibition was calculated as:

Inhibition (%) = (1-OD treated​/OD control​​) ×100.

The minimum inhibitory concentration (MIC) was defined as the lowest drug concentration exhibiting ≥90% inhibitory or complete absence of visible growth.

### 2.10 RNA extraction and quantitative RT‐PCR

Total RNA was extracted from exponential‐phase *L. acidophilus* cultures (untreated and treated: 50 and 100 µg/mL with PPIs) using the PureLink™ RNA Mini Kit (Thermo Fisher Scientific). First‐strand cDNA synthesis was performed with 2 µg RNA using the PrimeScript™ 1st Strand cDNA Synthesis Kit (Takara) according to manufactures’ protocols. qRT‐PCR reactions were prepared using GoTaq^®^ qPCR Master Mix (Promega) according to manufacture protocols and run on an ABI QuantStudio 5 system (Applied Biosystems, Canada) under the following conditions: 95 °C for 3 min; 40 cycles of 95 °C for 30 s, 60 °C for 30 s, and 72 °C for 30 s. Specificity was confirmed by melting curve analysis. The 16S rRNA gene served as internal control. Target genes included *nadH*, *pyrH*, *atpD*, *def*, *glmS*, and *murE* (**Supplementary Table 1**). PPI solutions were freshly prepared in order to minimize potential degradation under near-neutral culture conditions.

### 2.11 Statistical analysis

All quantitative analyses, including OD measurements, percentage inhibition, IC_50_ estimation, ΔΔCt calculation, and 2^-ΔΔCt^-based fold-change determination, were performed in R (version 4.5.2, 2025-02–28 ucrt) (RStudio Team, 2025). Data processing and summarization were conducted using the tidyverse suite, while IC_50_ values were derived using the drc package. Dose-response modelling and graphical visualizations were generated with ggplot2.

## 3 Results

### 3.1 Subtractive genomic approach to identify essential cytoplasmic non-homologous proteins

The core-genome analysis of *L. acidophilus* yielded 1,534 coding DNA sequences (CDSs), representing the conserved core proteome (**Supplementary Table 2**). Redundancy removal at a 70% identity threshold using the CD-HIT suite reduced the dataset to 1,532 non-redundant protein sequences. From this set, 279 proteins were predicted to be essential using the GEPTOP server. Subcellular localization analysis revealed that 233 of these essential proteins were localized in cytoplasm. Further homology screening against the human proteome identified 93 essential non-homologous cytoplasmic proteins, which may serve as potential therapeutic or probiotic targets.

Application of the multi-parameter scoring system enabled systematic classification of the predicted proteins into different confidence levels of drug target potential. In total, 40 proteins (43.01%) were identified as very high-confidence targets, 29 proteins (31.18%) as high-confidence, 15 proteins (16.12%) as moderate-confidence, and 9 proteins (9.67%) as low-confidence or non-targets (**Supplementary Figure 1**). This sorting demonstrates the robustness of the filtering approach in refining a broad protein dataset into a smaller subset of reliable and biologically relevant candidates suitable for downstream structural modelling and docking analyses. Comparable *in-silico* pipelines have reported that approximately 30-50% of proteins typically pass stringent multi-criteria prioritization, supporting the reliability and reproducibility of this strategy for identifying high-quality drug targets.

### 3.2 KEGG functional annotation and pathway enrichment

Functional annotation of the nominated proteins against the KEGG database demonstrated enrichment across multiple metabolic and cellular processes (**Supplementary Figure 2****;** **Supplementary Table 3**). The major category was genetic information processing comprised of 26 proteins (33.8%), followed by protein families involved in genetic processes with 19 proteins (24.7%). Among metabolic pathways, amino acid metabolism was considerably enriched (8 proteins; 10.4%), mostly from the lysine biosynthesis route. Nucleotide metabolism and other amino acid pathways each provided 4 proteins (5.2%), emphasizing their role in DNA/RNA synthesis and repair. Glycan biosynthesis and metabolism, notably peptidoglycan production, accounted for 3 proteins (3.9%), emphasizing cell wall integrity. Similarly, energy metabolism provided 3 proteins (3.9%), mostly engaged in oxidative phosphorylation. Additional hits were linked to carbohydrate metabolism, lipid metabolism, cofactors and vitamins. A modest subset (2 proteins) remained unclassified within genetic information processing.

Pathway-specific enrichment revealed four important functional categories: cell wall biosynthesis, nucleotide metabolism, amino acid biosynthesis, and energy metabolism (**Supplementary Table 3**). Peptidoglycan biosynthesis was dominated by MurA-MurF and Ddl, supported by GlmS and GalU for precursor supply, with CobS revealing linkage between cobalamin and glycan pathways. Nucleotide metabolism comprised NrdB (purine) and Cmk, Dut, and PyrH (pyrimidine), establishing an interconnected DNA/RNA maintenance component. Amino acid metabolism was led by the lysine (diaminopimelate) route, with MurE and MurF bridging lysine and cell wall synthesis. Energy metabolism was heightened by ATP synthase subunits (AtpA-AtpD-AtpC) and Pta, coupling carbon flux to ATP synthesis. Together, these pathways constitute substantial metabolic axes supporting bacterial survival and possible drug targets.

### 3.3 Protein-protein interaction network analysis

**Figure 2:**
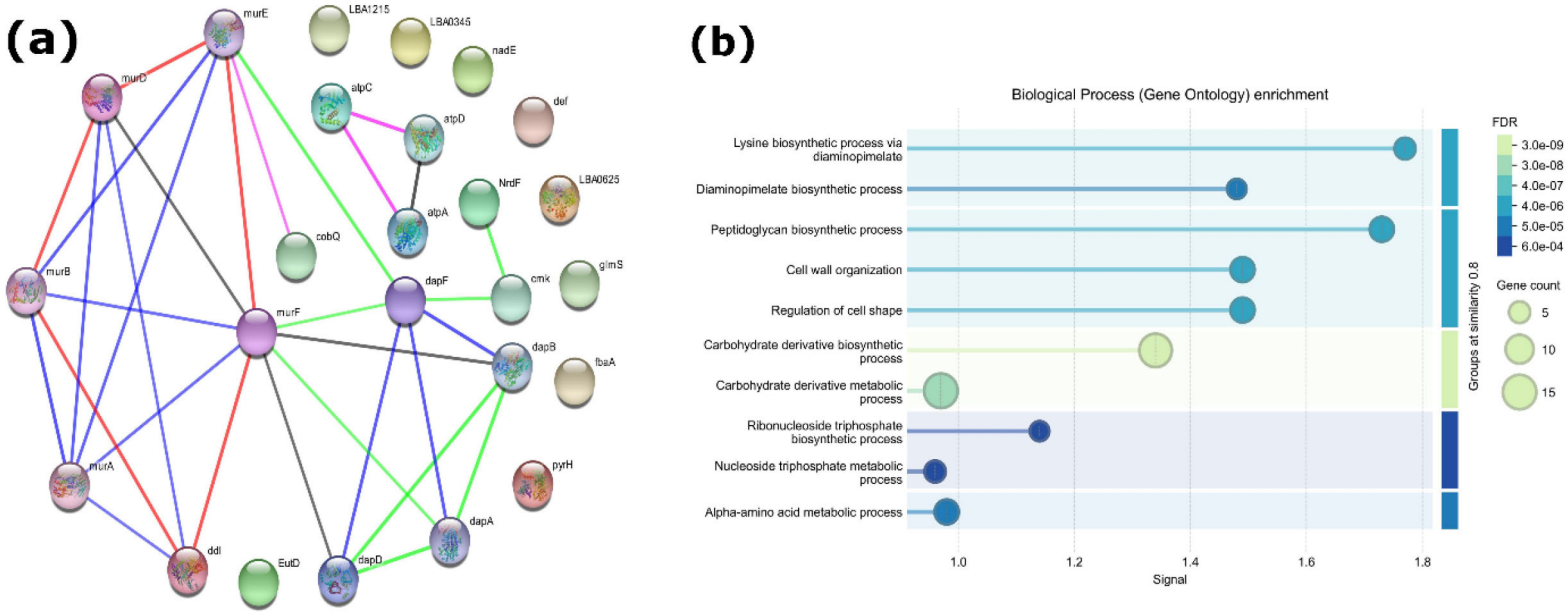
Protein interaction network and functional enrichment in *L. acidophilus*. **(a)** Protein-protein interaction network created with a confidence score ≥0.7, where nodes represent proteins and edges indicate functional relationships supported by experimental and computational data. Node size denotes connection, revealing hub proteins and functional modules. **(b)** Gene Ontology enrichment of network proteins demonstrating considerably enriched biological processes. Circle size denotes gene count, and colour intensity reflects statistical significance (FDR).

The analysis of the protein-protein interaction network for the selected proteins revealed 25 nodes interconnected by 31 edges, yielding an average node degree of 2.48 and a clustering coefficient of 0.523, which is substantially greater than anticipated (expected edges = 5; enrichment p = 2.07 × 10^-^¹^4^), signifying robust functional interconnectivity (**Figure 2a**). Functional enrichment demonstrated a notable overrepresentation of peptidoglycan biosynthesis, cell wall biogenesis and degradation, as well as lysine/diaminopimelate biosynthesis, amino acid metabolism, carbohydrate metabolism, and processes associated with cell division, ATP binding, and energy metabolism (**Figure 2b**).

Multiple hub proteins surfaced as pivotal nodes throughout the network. In peptidoglycan biosynthesis, MurA, MurE, MurF, and Ddl constitute a tightly interconnected cluster, underscoring their critical functions in precursor production and crosslinking. Other essential modules encompassed the lysine/diaminopimelate pathway (DapA, DapD, DapF) and the F_0_F_1_ ATP synthase complex (AtpA, AtpD, AtpC). Peripheral yet functionally essential proteins, including GlmS, Def, NadE, and PyrH, were found, facilitating amino sugar supply, protein maturation, NAD production, and nucleotide metabolism. Collectively, these data reveal a series of hub-associated and important metabolic proteins that create a highly linked network and provide promising antibacterial target candidates.

### 3.4 3D structural modelling and targets prioritization

The structural reliability of 14 selected proteins from *L. acidophilus* was assessed using AlphaFold2. All models demonstrated high confidence, with pLDDT values >90, confirming reliable predictions. Among these, DapA (96.98), AtpC (96.09), and DapD (95.93) scored best, while AtpD (90.71) was the lowest but still within the high-confidence range.

Thorough quality evaluation validation of DapD and Ddl structures suggested that DapD achieved an ERRAT score of 89.68 and a VERIFY3D score of 42.80, whereas Ddl exhibited ERRAT score of 90.22 and VERIFY3D score of 78.06. Ramachandran analysis indicated 90.5% (DapD) and 92.1% (Ddl) residues in preferred areas, suggesting structural reliability. However, fpocket analysis suggested poor druggability. DapD scored 0.316 with a druggability index of 0.340 and 75 alpha spheres, whereas Ddl scored 0.196 with an index of 0.005 and 38 alpha spheres. Since druggability scores <0.5 often imply low ligand-binding potential, both proteins were rejected from additional docking investigations despite satisfactory structural validation.

### 3.5 Structural validation of predicted protein models

The refined 3D protein structures were validated using ERRAT, VERIFY3D, Ramachandran plots, and ProSA Z-scores (**Supplementary Table 4**). All models displayed great overall quality, with ERRAT scores ranging from 95.65 (AtpC) to 99.57 (PyrH), substantially above the threshold of 50, demonstrating stable non-bonded atomic interactions. VERIFY3D further reinforced model integrity, with most proteins supporting >70% residues in favorable 3D-1D profiles. GatD revealed the greatest VERIFY3D score (99.56%), whereas AtpC got the lowest (58.90%) but remained acceptable. Ramachandran plots confirmed good stereochemical quality, with all proteins above 92% residues in favorable regions, specifically AtpC (98.5%), PyrH (97.5%), NadE (97.1%), and MurB (97.2%). ProSA Z-scores varied from -6.05 (AtpC) to -12.23 (GlmS), commensurate with experimentally solved structures of similar size. The most favorable scores were reported for GlmS (-12.23), MurA (-11.86), MurF (-11.59), AtpD (-11.36), and MurE (-11.01), showing good structural stability, whereas AtpC, Def, and NadE showed less unfavourable but acceptable values. Collectively, this exploration verified the high stereochemical and energetic quality of the protein models, while AtpC needed cautious interpretation due to inferior VERIFY3D and ProSA profiles.

FPocket was used to further evaluate the druggability of these verified models (**Supplementary Table 5**). High druggability indices (>0.95) were shown by a number of proteins, showing clearly delineated ligand-accessible binding sites. MurB (druggability = 1.000, score = 1.318, 142 alpha spheres, volume = 1236.23 Å³), NadE (1.000, 1.467, 165, 1580.32 Å³), MurE (0.997, 1.447, 146, 1228.33 Å³), and GlmS (0.996, 1.598, 106, 1422.93 Å³) established the most favorable profiles, suggesting their potential as significant therapeutic targets. MurA (0.996) and GalU (0.981) also revealed very druggable pockets, while MurF (0.978), AtpD (0.975), and PyrH (0.967) demonstrated moderately strong potential. By contrast, AtpC (0.754, 0.707, 30, 349.31 Å³), DapA (0.481, 0.803, 29, 572.98 Å³), and GatD (0.899, 0.849, 39, 435.28 Å³) displayed weaker druggability due to smaller or poorly defined binding sites. Overall, structural validation combined with FPocket analysis suggests that MurA, MurB, MurE, MurF, GlmS, GalU, NadE, Def,AtpD, and PyrH are the most promising candidates for structure-based drug discovery.

### 3.6 Molecular docking of proton pump inhibitors with druggable target proteins

Molecular docking was performed to evaluate the binding potential of six PPIs such as pantoprazole (4679), rabeprazole (5029), omeprazole (4594), lansoprazole (3883), dexlansoprazole (9578005), and esomeprazole (9568614) with key essential bacterial proteins. Docking interactions were assessed using MolDock scores, re-rank scores, and hydrogen bond (H-bond) energies, which reflect binding affinity and stability (**Table 1**; **Supplementary Table 6**).

**Table 1:** Best docking pose per bacterial protein with proton pump inhibitors.

Protein	PPI agents (PubChem compound ID)	MolDock score	Re-rank score	H-bond energy
MurA	Rabeprazole (5029)	-126.911	-91.749	-5.39
	Pantoprazole (4679)	-124.85	-95.3137	-11.0866
MurB	Rabeprazole (5029)	-153.866	-120.433	-5.99
	Omeprazole (4594)	-132.286	-70.0527	-6.4281
MurE	Pantoprazole (4679)	-153.146	-126.680	-3.57
	Esomeprazole (9568614)	-150.299	-126.264	-3.12651
GlmS	Rabeprazole (5029)	-129.901	-102.061	-6.77
	Pantoprazole (4679)	-129.57	-104.244	-4.95789
NadE	Pantoprazole (4679)	-147.969	-120.438	-4.98
	Dexlansoprazole (9578005)	-149.242	-107.481	-2.30328
AtpD	Pantoprazole (4679)	-133.700	-97.178	-11.39
PyrH	Rabeprazole (5029)	-127.255	-92.096	-6.43
	Pantoprazole (4679)	-118.117	-85.5339	-7.82657
Def	Lansoprazole (3883)	-143.93	-107.72	-4.91171
	Pantoprazole (4679)	-133.531	-100.755	-5.79

#### 3.6.1 Targeting Cell wall biosynthesis

**Figure 3:**
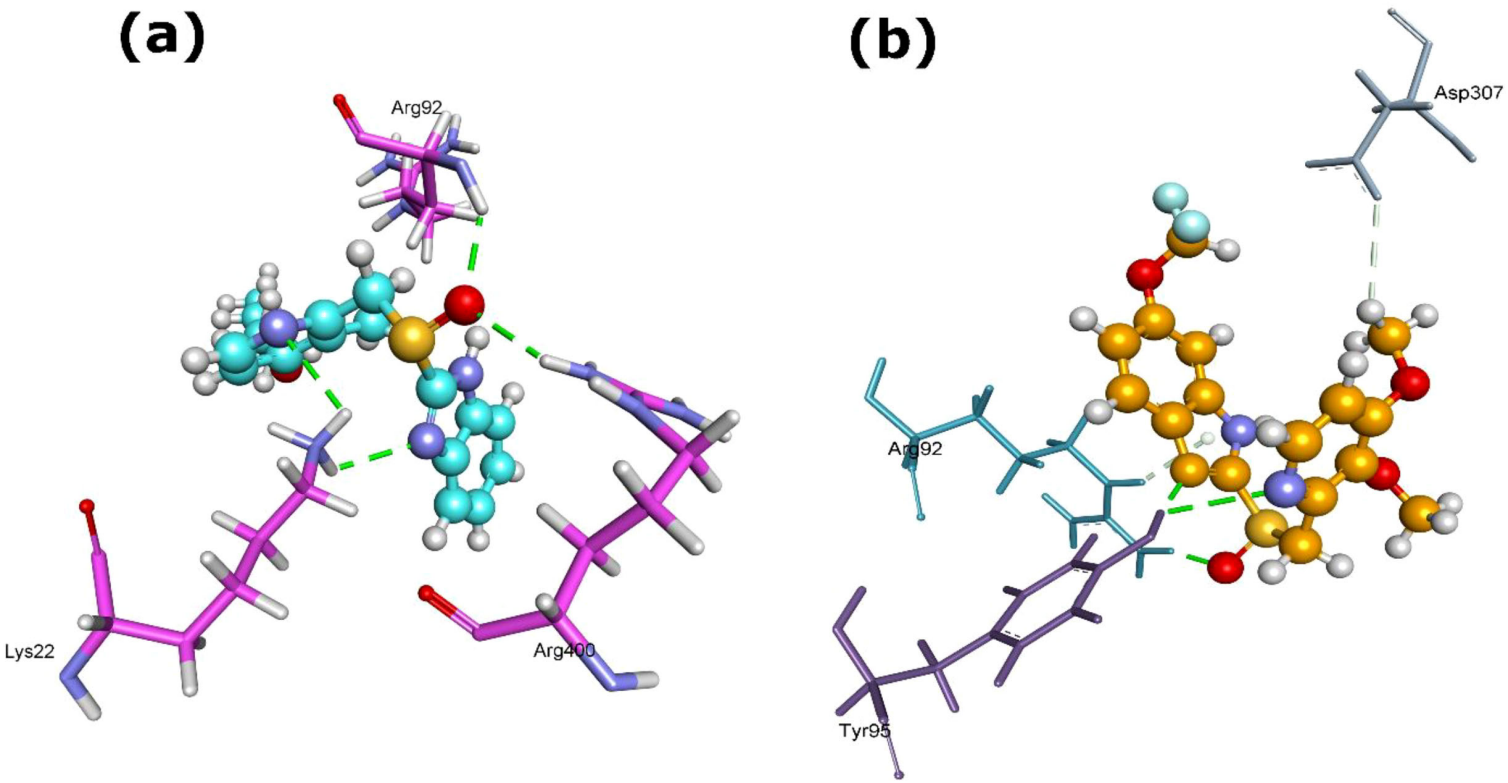
3D structures of MurA protein complexed with **(a)** rabeprazole (5029) and **(b)** pantoprazole (4679). Dash green line represents the H-bonds with the residues of MurA protein.

The Mur protein family, central to peptidoglycan biosynthesis, emerged as strong binding targets for several PPIs (**Supplementary Table 6**). Among the studied PPIs, rabeprazole demonstrated effective binding with MurA, forming four hydrogen bonds (Lys22 [2×], Arg92, Arg400) and yielding MolDock and Re-rank scores of -126.911 and -91.749, respectively (**Table 1**; **Figure 3a**). Pantoprazole forming stronger hydrogen-bond interactions (H-bond energy -11.1) with the MurA protein residues Arg92, Tyr95 [2×], and Asp307 (**Figure 3b**).

**Figure 4:**
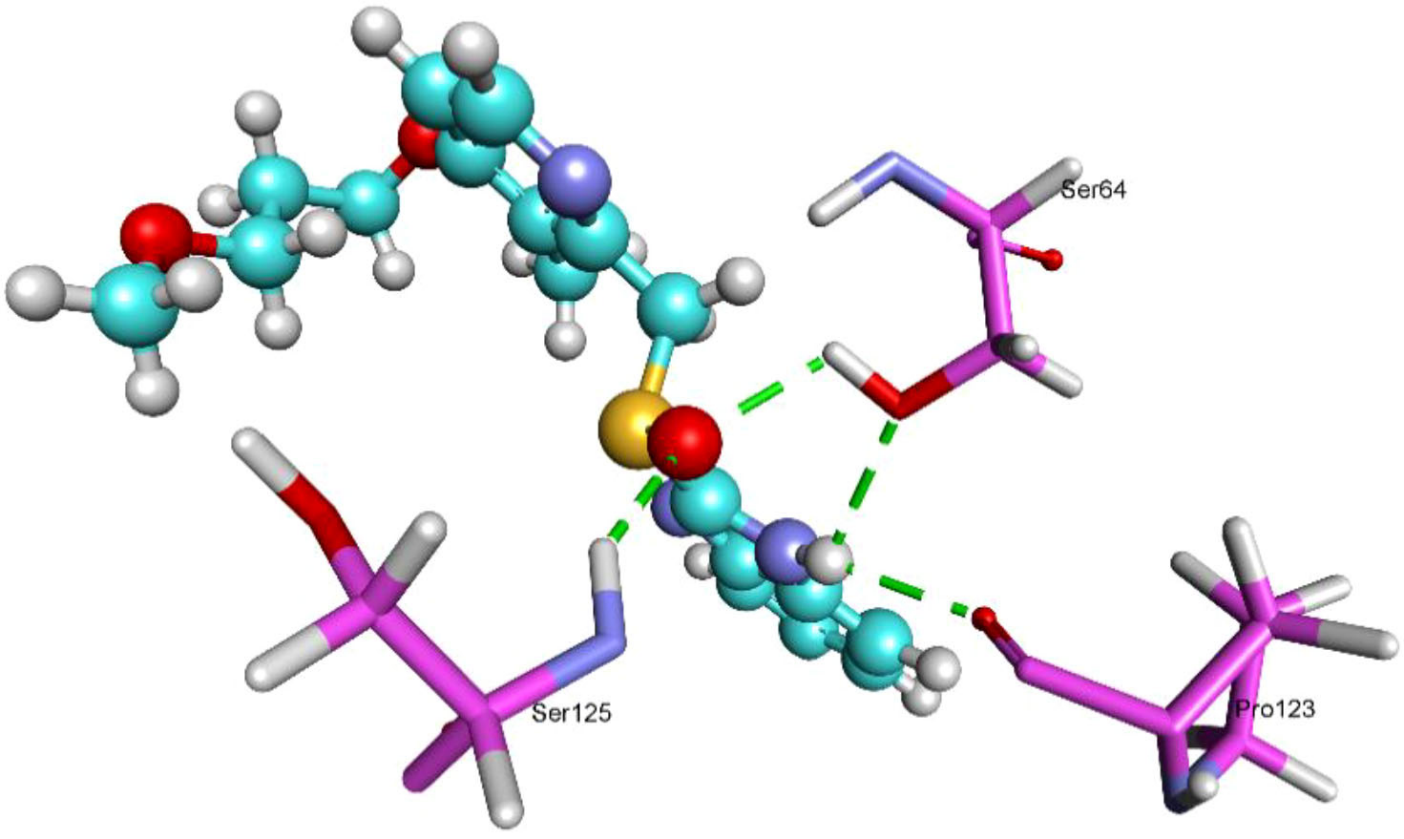
3D structures of MurB protein complexed with rabeprazole (5029). Dash green line represents the H-bonds with the residues of MurB protein.

MurB exhibited the strongest interaction with rabeprazole (MolDock -153.9; re-rank -120.4) creating four H-bonds with Ser64[2×], Pro123, and Ser125, suggesting a highly stable binding conformation (**Figure 4**). while omeprazole showed comparatively weaker but still favorable binding (**Table 1**).

**Figure 5:**
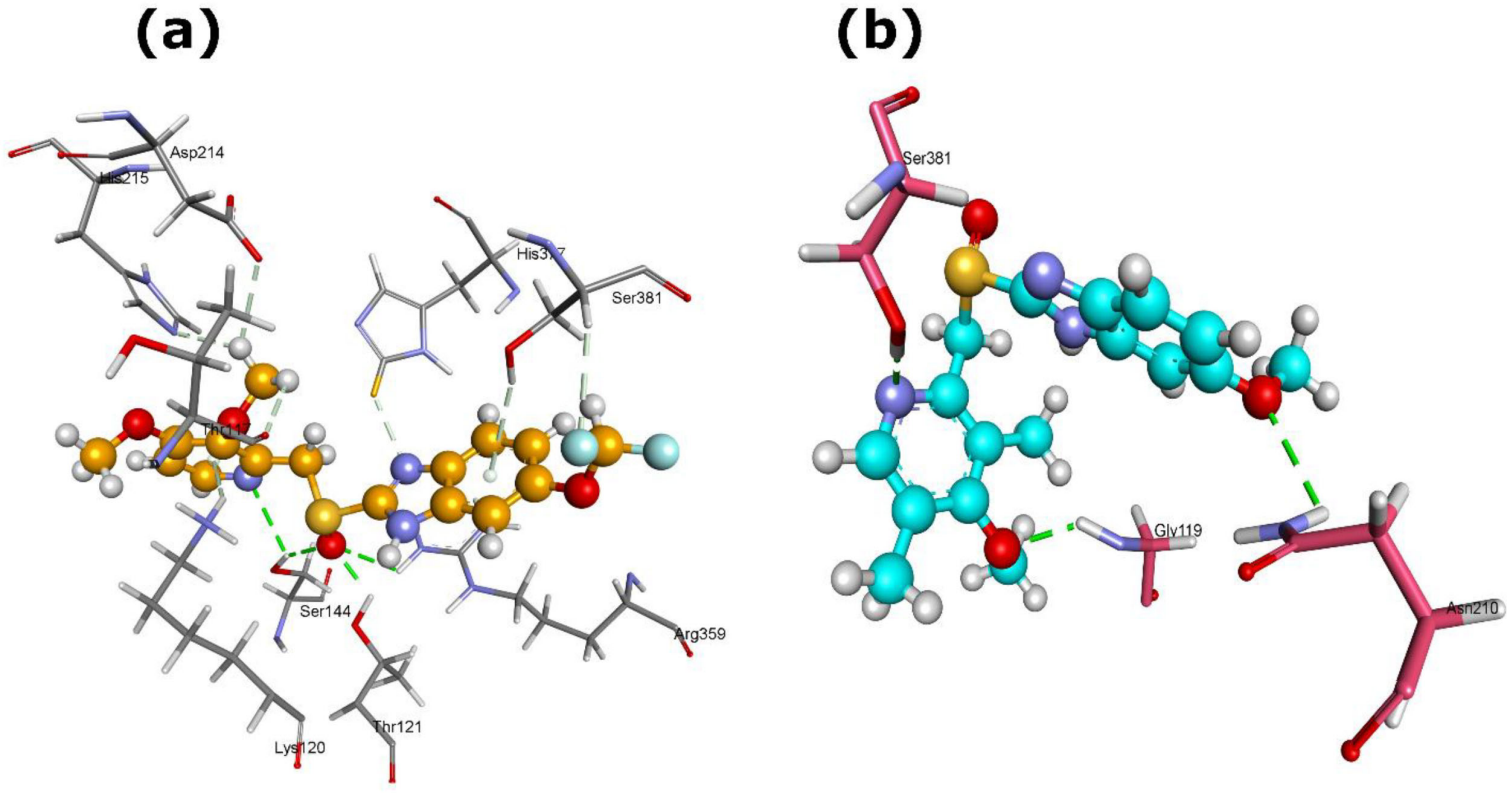
3D structures of MurE protein complexed with **(a)** pantoprazole and **(b)** esomeprazole (9568614). Dash green line represents the H-bonds with the residues of MurE protein.

In case of MurE protein, among the other PPIs, pantoprazole and esomeprazole exhibited nearly equivalent and highly favorable docking scores, with pantoprazole showing the best overall interaction (MolDock -153.1; re-rank -126.7), stabilized by four H-bonds with Ser144 [2×], Thr121, and Arg359 (**Figure 5a**). However, esomeprazole involved three H-bonds with Gly119, Asn210, and Ser381 of MurE protein (**Figure 5b**), indicating that this protein is a high susceptible target for PPI binding.

**Figure 6:**
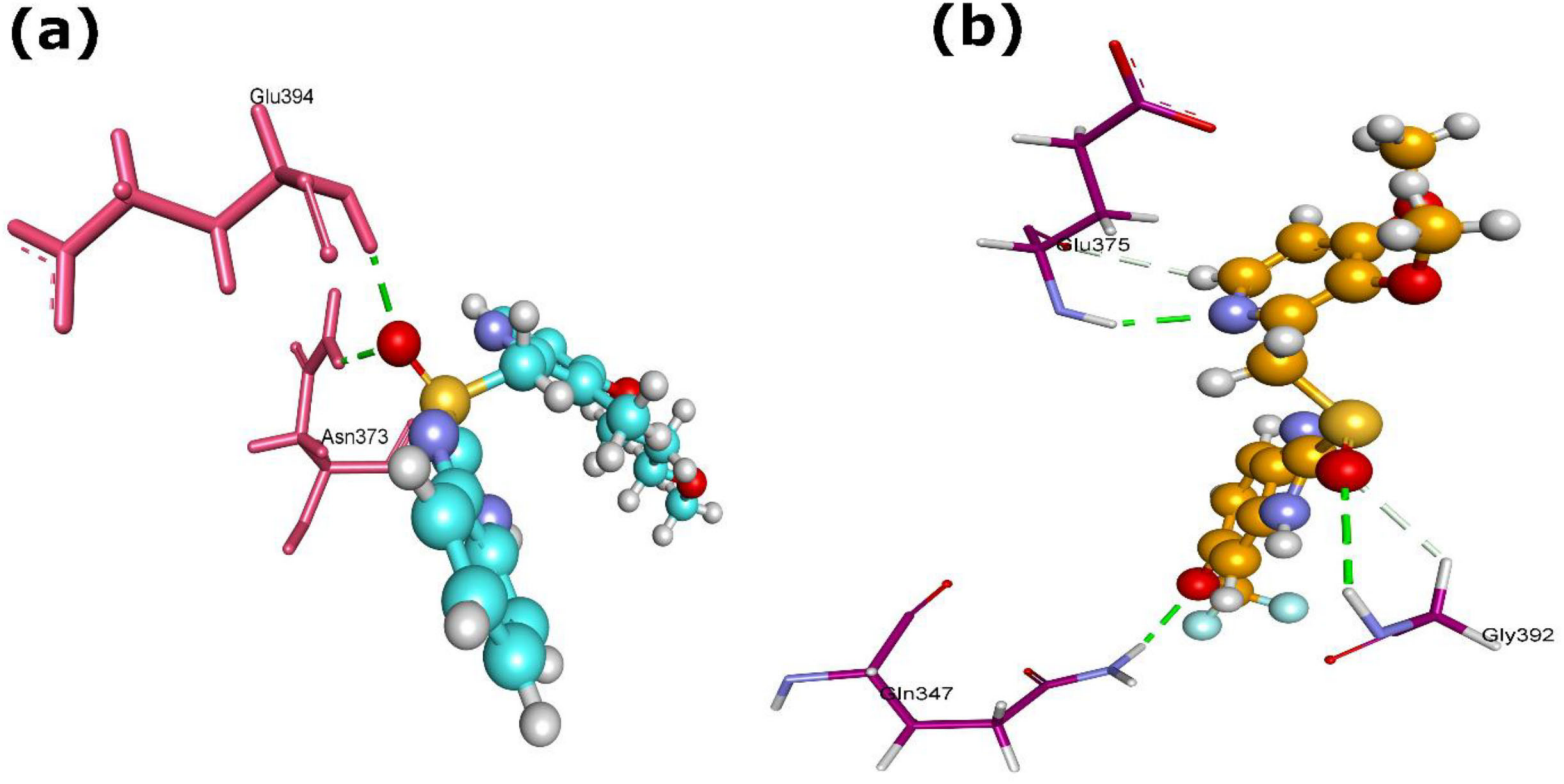
3D structures of GlmS protein complexed with **(a)** rabeprazole (5029) and (b) pantoprazole (4679). Dash green line denotes the H-bonds with the residues of GlmS protein.

In precursor-supply pathways, GlmS showed notable interaction with rabeprazole and pantoprazole, characterized by favorable docking scores, re-rank scores and hydrogen bond energies (**Table 1**). The GlmS-rabeprazole complex formed two H-bonds with residues Glu394 and Asn373 (**Figure 6a**), while the GlmS-pantoprazole complex generated three H-bonds with residues Gln347, Glu375, and Gly392 (**Figure 6b**).

These results suggest that PPIs, specifically rabeprazole and pantoprazole, may interact with enzymes involved in peptidoglycan formation in *L. acidophilus*, consequently affecting bacterial cell-wall integrity. In addition to Mur enzymes, GlmS emerges as a potential off-target for PPI interaction. Given its essential involvement in amino-sugar metabolism and peptidoglycan precursor production, suppression of GlmS could further disrupt cell-wall biosynthesis. Collectively, these interactions support a multi-target inhibitory effect of PPIs within the Mur enzyme family and associated precursor-supply pathways.

#### 3.6.2 Targeting nucleotide metabolism and ATP synthesis

**Figure 7:**
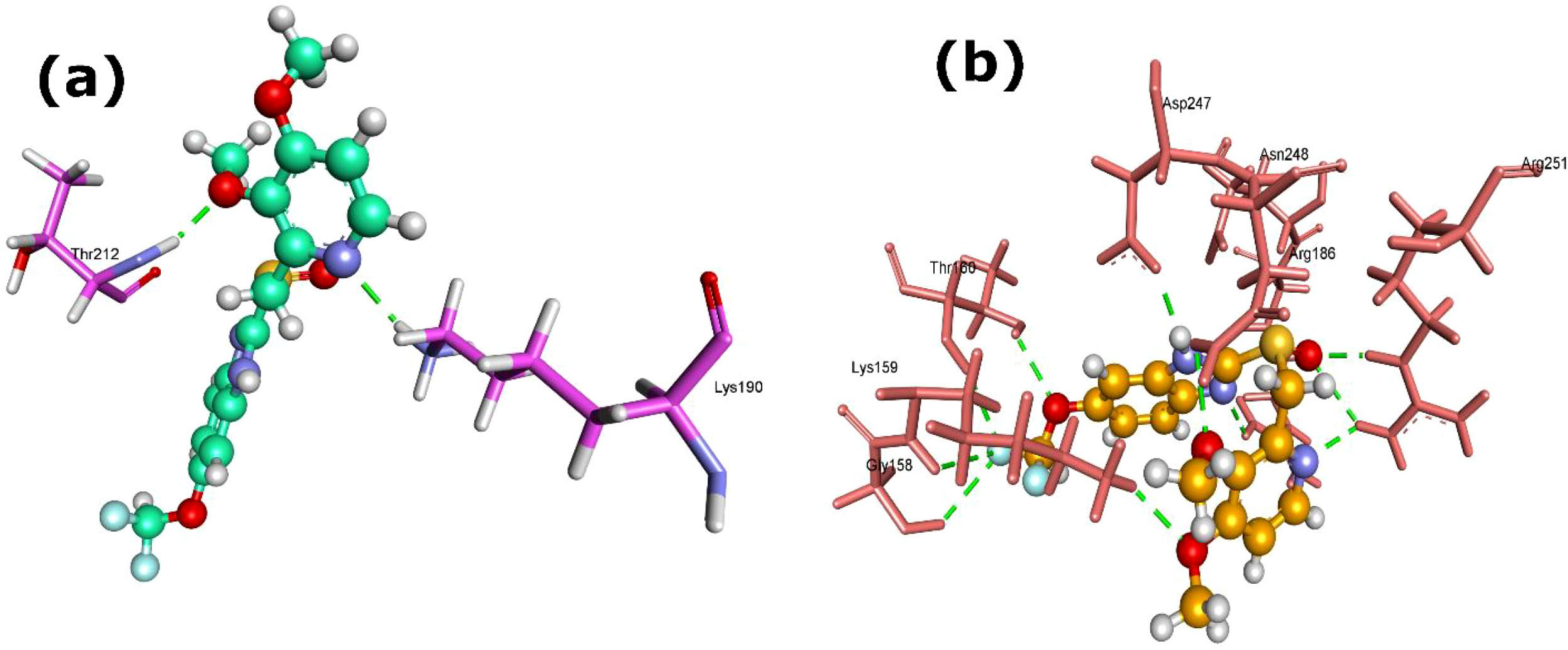
3D structures of pantoprazole (4679) complexed with **(a)** NadE, and **(b)** AtpD proteins. Dash green line represents the H-bonds with the protein residues.

Within nucleotide metabolism, pantoprazole and dexlansoprazole showed potent interactions with NadE, the NAD synthetase (**Table 1** and **Figure 7**). Pantoprazole (4679) achieved a MolDock score of -147.97, a highly favorable Re-rank score of -120.44, and an H-bond contribution of -4.98, forming two H-bonds with Lys190 and Thr212 (**Figure 7a**). Dexlansoprazole (9578005) recorded the lowest MolDock score (-149.24) but a comparatively weaker Re-rank score (-107.48); it stabilized through three H-bonds with Asp214, Leu215, and Lys262. Rabeprazole (5029) also interacted with NadE (MolDock -147.25; Re-rank -101.86; H-bond -1.81), forming two H-bonds with Ser49 and Arg143. These results indicate that pantoprazole is predicted to have the most stable binding pose despite dexlansoprazole achieving a slightly lower MolDock score.

Among the other PPIs, pantoprazole (4679) showed strong binding affinity with AtpD, a subunit of ATP synthase. The docking complex exhibited a MolDock score of -133.70 and a Re-rank score of -97.18, with a significant H-bond energies of -11.39, reflecting extensive stabilization through polar interactions (**Table 1**). Pantoprazole formed eleven H-bonds with key residues, including Gly158 [2×], Lys159, Thr160 [2×], Arg186, Asp247, Asn248, and Arg251 [3×] (**Figure 7b**). Such strong interactions suggest that pantoprazole may potentially interfere with ATP synthase activity, thereby compromising energy metabolism in *L. acidophilus*.

**Figure 8:**
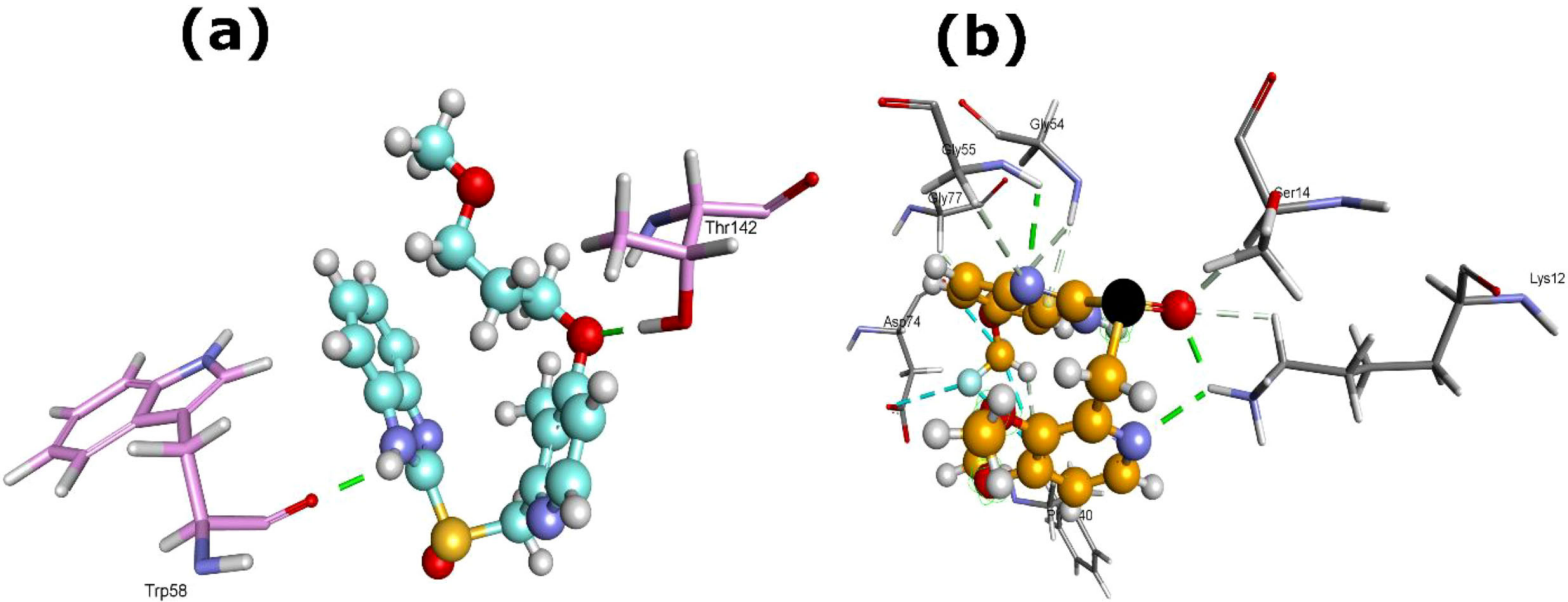
3D structures of PyrH protein complexed with **(a)** rabeprazole (5029) and **(b)** pantoprazole (4679). Dash green line denotes the H-bonds with the residues of PyrH protein.

For PyrH, rabeprazole exhibited the strongest binding, with a MolDock score of -127.26, Re-rank score of -92.10, and an H-bond contribution of -6.43. It generated two H-bonds with the protein residues Trp58 and Thr142 (**Table 1**; **Figure 8a**). Additionally, the interaction of PyrH and pantoprazole generated MolDock score of -118.117, Re-rank score of -85.53, and an H-bond contribution of -7.83. It created three H-bonds with the protein residues Lys12 [2x] and Gly55 (**Table 1**; **Figure 8b**). The affinities for PyrH were less pronounced than those observed for NadE, suggesting stronger potential interaction of PPIs with enzymes involved in NAD biosynthesis than on pyrimidine metabolism.

#### 3.6.3 Targeting protein synthesis enzymes

**Figure 9:**
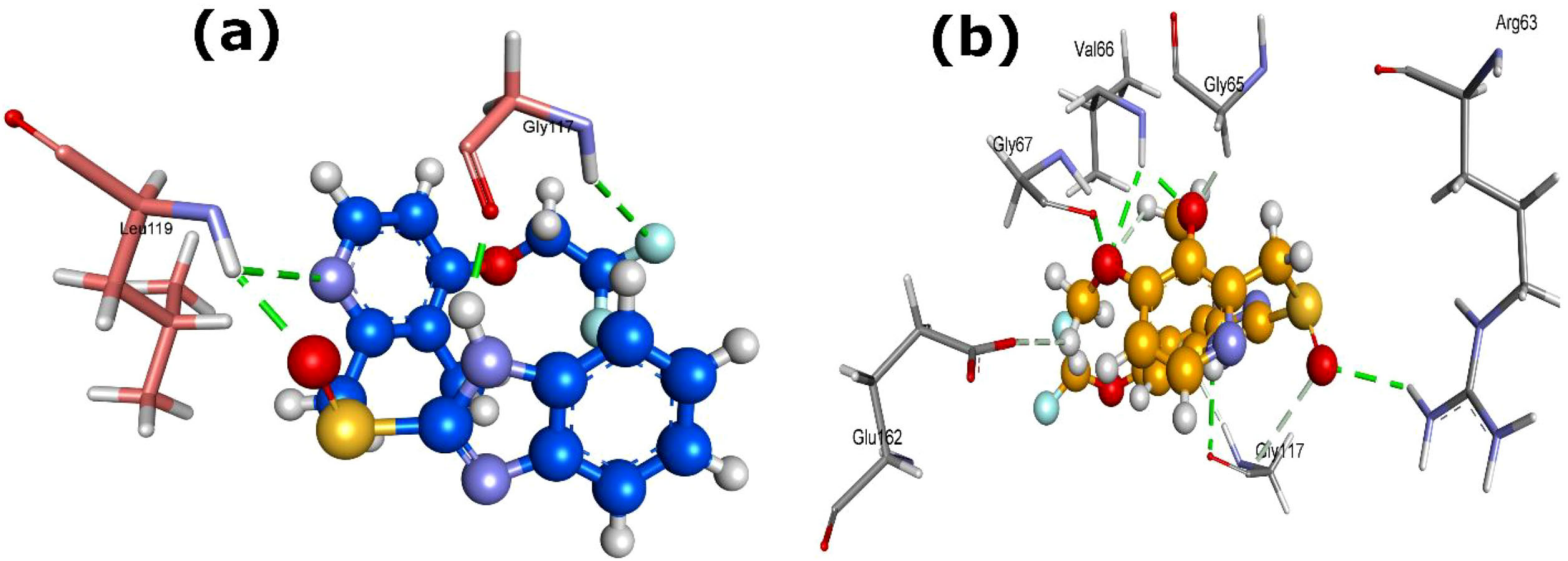
3D structures of Def protein complexed with **(a)** lansoprazole (3883) and **(b)** pantoprazole (4679). Dash green line signifies the H-bonds with the residues of Def protein.

In the case of peptide deformylase (Def), lansoprazole (3883) displayed the strongest interaction (MolDock -143.93; Re-rank -107.72; Hbond energies -4.91), forming four H-bonds with Gly117 [2x] and Leu119 [2x] (**Table 1**; **Figure 9a**). Pantoprazole also exhibited stable binding with Def (MolDock -133.53; Re-rank -100.76; Hbond energies -5.79), creating five H-bonds with Arg63, Val66 [2x], Gly67, and Gly117 (**Figure 9b**). These findings suggest that lansoprazole and pantoprazole may interfere with the deformylation process of nascent peptides, suggesting Def as another critical off-target for PPI-mediated suppression.

Among the other PPIs, pantoprazole revealed broad-spectrum binding across cell wall, energy, and metabolic targets, including MurA, MurE, GlmS, NadE, AtpD, PyrH, and Def, while rabeprazole specifically targeted important peptidoglycan and nucleotide biosynthesis enzymes (MurA, MurB, GlmS, and PyrH). When combined, these docking results offer compelling *in-silico* evidence that rabeprazole and pantoprazole are the most effective PPIs in terms of off-target interactions, supporting a mechanistic connection between PPI exposure and reduced bacterial cell fitness and integrity in *L. acidophilus*.

### 3.7 Molecular dynamics insights into high-affinity protein-PPI binding

To ascertain the dynamic stability of off-target complexes formed by PPIs with key essential bacterial proteins, MD simulations were performed for complexes bound to pantoprazole and rabeprazole. These two PPIs were selected from six tested target proteins due to their consistently strong docking performance against MurE, GlmS, AtpD, NadE, PyrH, and NadH, which are essential for cell wall biosynthesis, energy metabolism, NAD homeostasis, and nucleotide synthesis, as indicated by highly negative MolDock and re-rank scores, favorable H-bonding energies, and strong interaction profiles. To further evaluate the stability and dynamic behaviour of these protein-ligand complexes during simulation, several structural parameters were analysed from the MD trajectories. The RMSD was used to assess the overall conformational stability of the protein backbone and to determine whether the complexes maintained structural equilibrium over the simulation period. RMSF provided insights into residue-level flexibility and helped identify localized structural fluctuations induced by ligand binding. The Rg was calculated to evaluate the compactness of the protein structure and to determine whether ligand binding induced any global structural expansion or compaction. In addition, H-bond analysis was performed to monitor the persistence of intermolecular interactions between the ligands and key protein residues, which contributes to the stabilization of the complexes throughout the simulation. Collectively, these complementary metrics provide a comprehensive evaluation of the structural stability and dynamic behavior of the protein-ligand complexes during the MD simulation period.

#### 3.7.1 MD simulation of MurE and PPI bound MurE complexes

**Figure 10:**
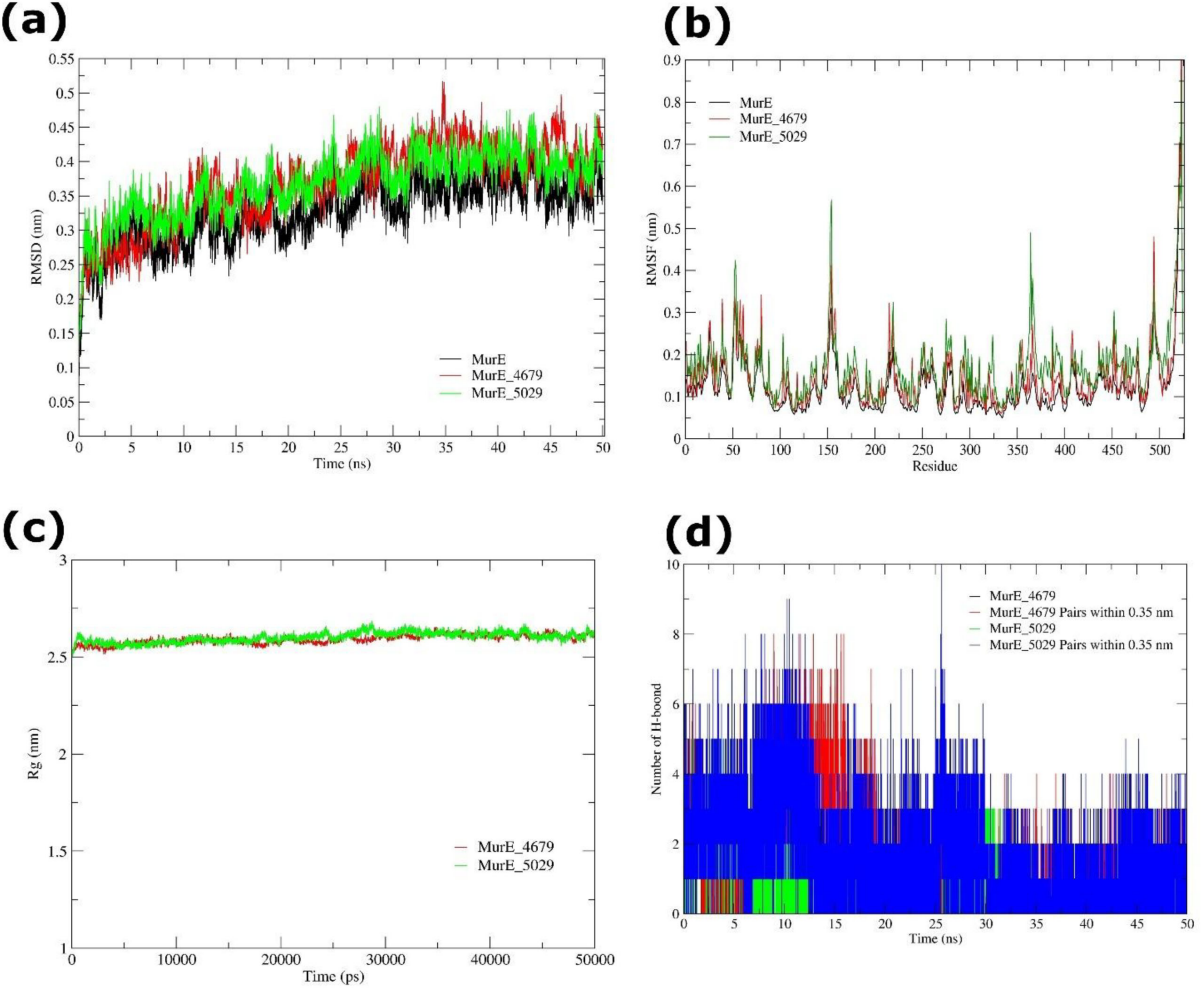
MD simulation of MurE (apo and ligand-bound) systems over 50 ns. **(a)** RMSD trajectories of MurE apo (black), MurE-4679 complex (red), and MurE-5029 complex (green), showing structural stability during the simulation. **(b)** RMSF profiles of MurE apo (black), MurE-4679 complex (red), and MurE-5029 complex (green), highlighting residue-level flexibility and conformational fluctuations. **(c)** Rg analysis of MurE-4679 (red), and MurE-5029 (green) complexes. **(d)** Number of intramolecular H-bond interactions formed by MurE-4679, and MuE-5029 complexes.

The overall structural stability of MurE, RMSD profiles were tracked throughout a 50 ns simulation for the apo protein and its complexes with pantoprazole (MurE-4679) and rabeprazole (MurE-5029). The apo MurE protein attained equilibrium at around 9.8 ns, exhibiting a plateau average RMSD of 0.334 nm, so confirming stability following initial equilibration. Both ligand-bound systems demonstrated analogous stabilization patterns, with slight variations in equilibration durations and RMSD values. The MurE-4679 complex equilibrated swiftly at ~1.3 ns, sustaining a mean RMSD of 0.368 ± 0.059 nm (mean ± Standard Deviation) across 5001 trajectory frames (0.01 ns interval). The low SE (standard error) (8.47 × 10^-4^) highlights the stability of the trajectory mean. Statistical distribution analysis indicated moderate negative skewness (cum.3 = -0.407) and near-Gaussian kurtosis (cum.4 = 0.052), indicating that most conformations clustered closely around the mean with few extreme deviations. Conversely, the MurE-5029 complex achieved stabilization later, approximately at 9.8 ns, exhibiting an average RMSD of 0.368 ± 0.046 nm. A smaller relative standard error (SE) of 2.95 × 10^-4^ verified trajectory convergence. The distribution displayed negative skewness (cum.3 = -0.571) and slight positive kurtosis (cum.4 = 0.601), demonstrating a tightly packed RMSD profile with few outliers. The data together demonstrate that ligand contact did not destabilize MurE but rather generated slight conformational modifications essential for ligand accommodation (**Figure 10a**).

Local flexibility of MurE was analysed by RMSF of Cα atoms. In the MurE-4679 complex, RMSF values ranged from 0.05-0.92 nm, with a mean of 0.12 ± 0.073 nm. Residues with elevated fluctuations (>0.19 nm, measured as mean ± 1 SD) were restricted to surface-exposed loops (residues 25-27, 51-54, 56-61, 80, 151-158, 160, 215, 219, 490-499, and 515-523), whereas the protein core remained inflexible. These data show that pantoprazole binding maintains backbone stability while retaining loop flexibility to allow ligand accommodation. The MurE-5029 complex displayed slightly lower variations (range: 0.055-0.786 nm, mean = 0.148 nm), with flexible areas limited to residues 51-55, 151-155, 218-219, 363-367, 494-495, and 511-523. Notably, variations in the N-terminal loop (25-27) and loop 56–61 were decreased in the MurE-5029 complex relative to MurE-4679, suggesting rabeprazole imparted stabilizing effects on these areas. However, the C-terminal loop (511-523) remained fundamentally dynamic in both complexes, revealing a naturally flexible area. Overall, rabeprazole gave more local stability compared to pantoprazole, notably in loop regions crucial for ligand recognition (**Figure 10b**). Rg analysis was utilized to examine global compactness of MurE. The MurE-4679 complex maintained an average Rg of 2.59 ± 0.02 nm, while the MurE-5029 complex showed a nearly equal value of 2.60 ± 0.03 nm. The modest changes throughout both paths revealed that MurE kept its compact tertiary fold following ligand binding, consistent with RMSD and RMSF profiles (**Figure 10c**).

The MurE-4679 complex produced an average of 0.96 ± 0.99 H-bonds during the simulation, with 1.55 ± 1.63 donor-acceptor pairs seen within a 0.35 nm limit. The distribution diverged modestly from Gaussian (cumulants 0.472 and -0.075), consistent with dynamic yet recurring H-bond interactions. In comparison, the MurE-5029 complex exhibited fewer hydrogen bonds (0.44 ± 0.59 on average), with 1.34 ± 1.02 donor-acceptor pairs found at the 0.35 nm threshold. The mildly skewed distribution (cumulants 0.699/0.435 for H-bonds; 0.387/0.073 for pairs) showed transient and less persistent H-bonding patterns. Despite less persistent H-bonds, rabeprazole stabilized loop dynamics (as revealed in RMSF), suggesting its stabilizing impact may stem more from hydrophobic contacts and steric complementarity rather than stringent hydrogen bonding (**Figure 10d**).

The MD simulations indicated that pantoprazole (4679) and rabeprazole (5029) did not impact the global stability or compactness of MurE. Both ligands caused small conformational modifications, with rabeprazole imparting higher local stability of flexible loops despite reduced H-bond persistence. These results indicate that both ligands boost MurE stability while preserving the structural flexibility required for ligand accommodation, suggesting that MurE may represent a potential interaction target of PPIs.

#### 3.7.2 MD simulation of GlmS and PPI bound GlmS complexes

**Figure 11:**
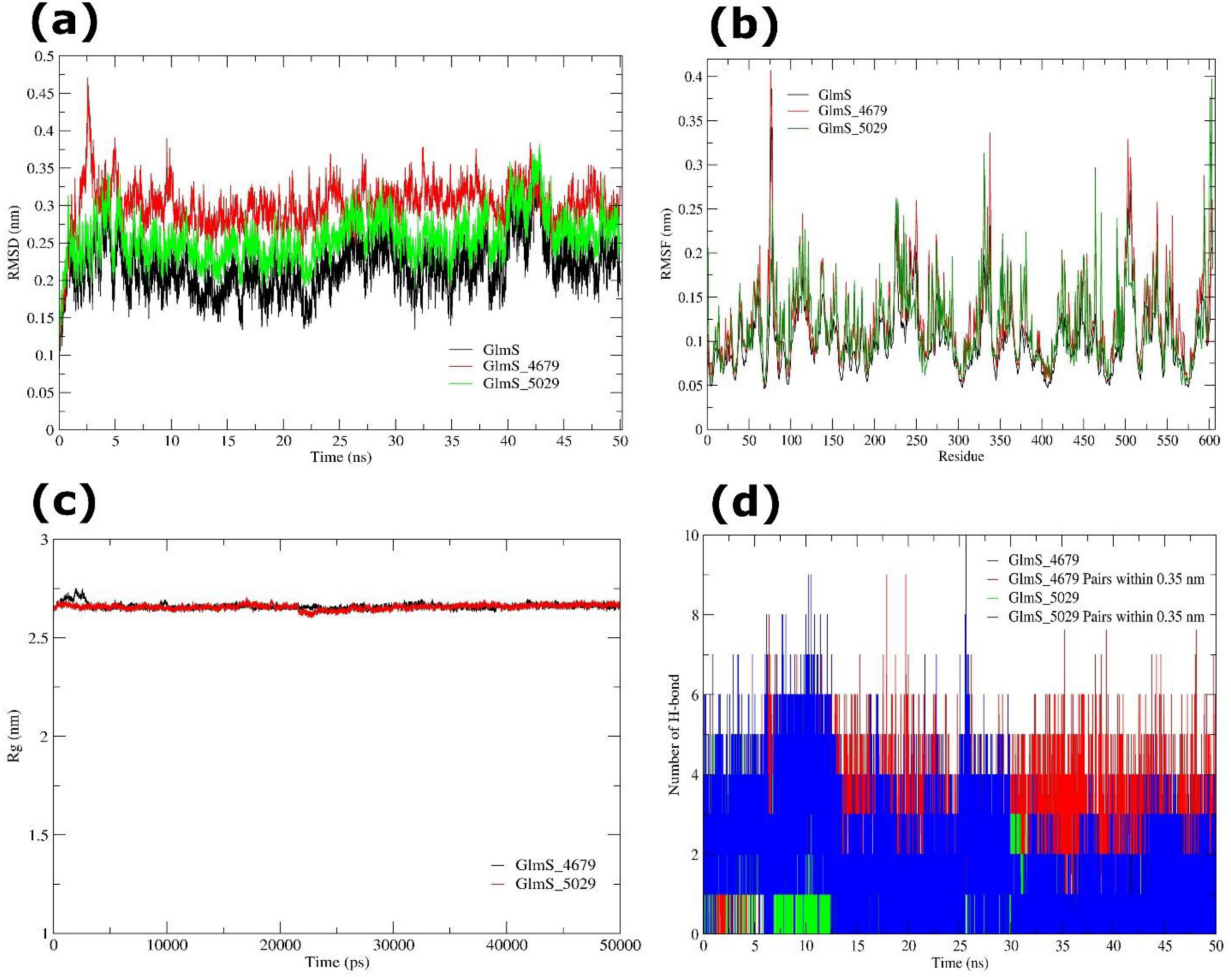
MD simulation of GlmS (apo and ligand-bound) systems during 50 ns. **(a)** RMSD trajectories of GlmS apo (black), GlmS-4679 complex (red), and GlmS-5029 complex (green), indicating structural stability during the simulation. **(b)** RMSF profiles of GlmS apo (black), GlmS-4679 complex (red), and GlmS-5029 complex (green), revealing residue-level flexibility and conformational variations. **(c)** Rg analysis of GlmS-4679 (black), and GlmS-5029 (red) complexes. **(d)** Number of intramolecular H-bond interactions formed by GlmS-4679, and GlmS-5029 complexes.

Backbone RMSD profiles were evaluated to determine the global stability of GlmS in its apo state and in complexes with pantoprazole (GlmS-4679) and rabeprazole (GlmS-5029). The apo GlmS protein exhibited a mean RMSD of 0.262 ± 0.033 nm throughout 5001 trajectory frames (dt = 0.01 ns), demonstrating a stable conformation with relatively small changes from the beginning structure. The GlmS-4679 complex stabilized swiftly within ~1.2 ns and maintained a mean RMSD of 0.300 ± 0.032 nm, while the GlmS-5029 complex equilibrated significantly later, at ~1.6 ns, with a mean RMSD of 0.256 ± 0.031 nm. In all three systems, variations were minor and within acceptable ranges for stable proteins. Skewness and kurtosis investigations further indicated that most conformations remained near to the mean, with little severe fluctuations. These findings reveal that both ligand-bound systems, comparable to the apo protein, swiftly reached equilibrium and kept stable trajectories during the 50 ns simulation (**Figure 11a**).

The apo GlmS protein exhibited a mean RMSF of 0.100 ± 0.039 nm, with localized variations confined to loop and solvent-exposed areas. In the GlmS-4679 complex, average RMSF values were comparable (0.100 nm, range: 0.046-0.386 nm), with higher flexibility at residues 59-61, 75-78, 110-115, 224-228, 242-251, and 330-338, largely surface loops. The GlmS-5029 complex revealed a somewhat larger mean RMSF of 0.119 ± 0.047 nm (range: 0.050-0.398 nm), with flexible segments at residues 76-79, 136-139, 224-229, 330-332, and 599-604. Further, in both ligand-bound systems, the protein core remained stiff, showing that ligand binding did not impact overall structural integrity. Instead, fluctuations were restricted to dynamic loops, highlighting that ligand-induced flexibility may play a role in altering functional movements of GlmS (**Figure 11b**). To assess global compactness, Rg was determined for apo and ligand-bound systems. The apo GlmS protein exhibited a mean Rg of 2.666 ± 0.015 nm, which was consistent to GlmS-4679 (2.660 ± 0.015 nm) and GlmS-5029 (2.656 ± 0.013 nm). Although slight secondary fluctuations were identified (2.24-1.89 nm), these did not suggest considerable unfolding or loss of compactness. Overall, the analysis indicated that both apo and ligand-bound systems kept a compact and stable fold along the 50 ns trajectory (**Figure 11c**).

The GlmS-4679 complex established a mean of 2.11 ± 0.65 H-bonds over the simulation, with 2.87 ± 1.35 donor-acceptor pairs found within a 0.35 nm threshold. The near-Gaussian distribution (cumulants 0.145 and 0.519) revealed stable hydrogen bonding patterns that facilitated ligand retention. In contrast, the GlmS-5029 complex generated fewer durable hydrogen bonds, averaging 0.86 ± 0.85, with 1.63 ± 1.46 donor-acceptor pairs found within the cutoff. Distribution analysis (cumulants 0.536/0.162 for H-bonds and 0.511/0.116 for pairs) found small variations, corresponding with weaker and more transitory hydrogen bonding (**Figure 11d**).

Together, RMSD, RMSF, Rg, and H-bond analyses reveal that pantoprazole (4679) and rabeprazole (5029) bind effectively to GlmS without disturbing its global fold. Pantoprazole displayed more consistent H-bond retention, but rabeprazole caused significantly more local flexibility, notably in loop areas. These findings ensure that both ligands preserve the structural stability of GlmS while marginally changing local dynamics, potentially impacting binding affinity and functional activity.

#### 3.7.3 MD simulation of AtpD and PPI bound AtpD complexes

**Figure 12:**
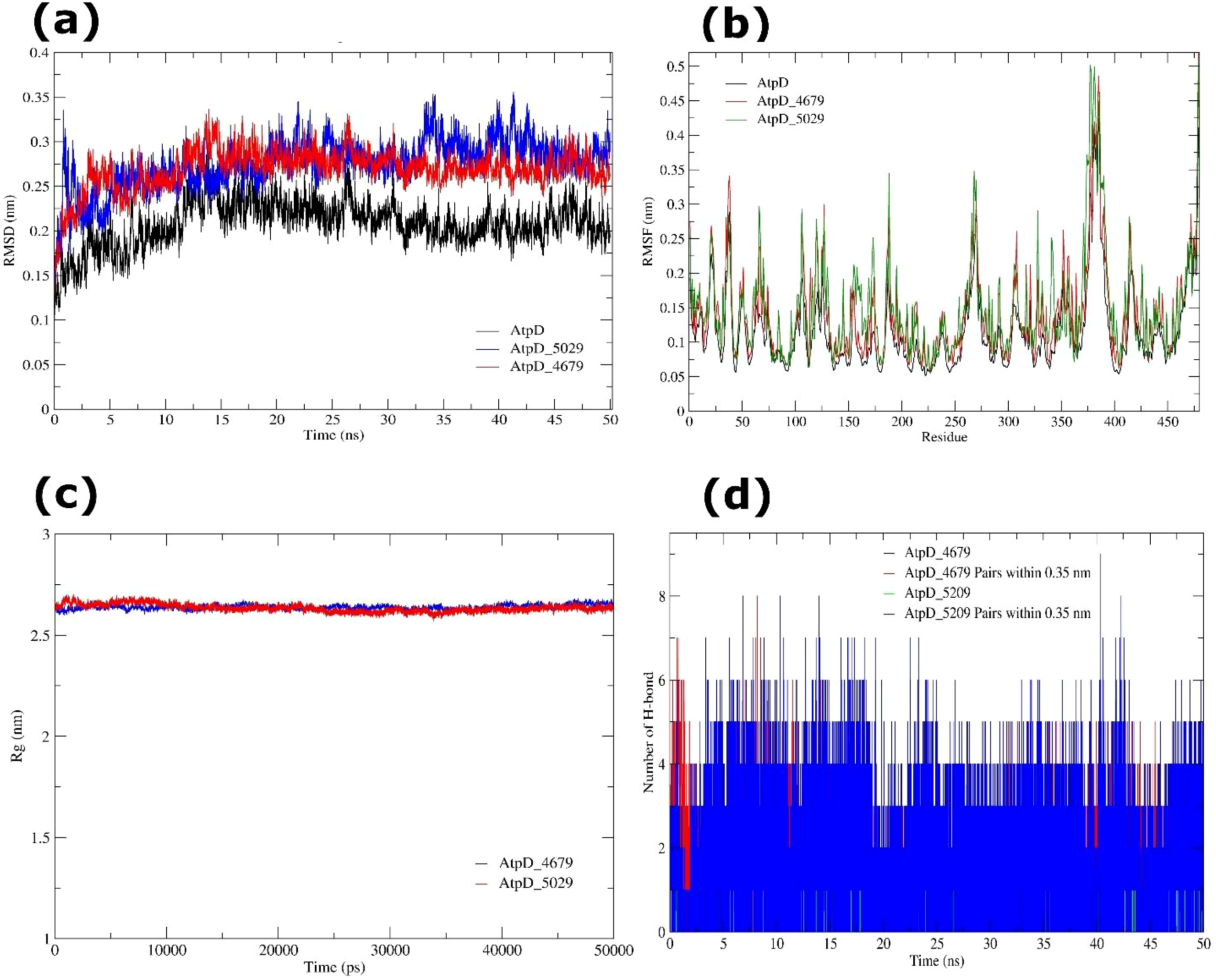
MD simulation of AtpD (apo and ligand-bound) systems over 50 ns. **(a)** RMSD trajectories of AtpD apo (black), AtpD-4679 complex (red), and AtpD-5029 complex (blue), showing structural stability during the simulation. **(b)** RMSF profiles of AtpD apo (black), AtpD-4679 complex (red), and AtpD-5029 complex (green), highlighting residue-level flexibility and conformational fluctuations. **(c)** Rg analysis of AtpD-4679 (blue), and AtpD-5029 (red) complexes. **(d)** Number of intramolecular H-bond interactions formed by AtpD-4679, and AtpD-5029 complexes.

The structural stability of AtpD was investigated by backbone RMSD profiles. The apo protein stabilized swiftly at ~3.03 ns, attaining a mean plateau RMSD of 0.208 ± 0.026 nm, suggesting a stable equilibrium with only minimal fluctuations. The low SE (3.66 × 10^-4^) supported the dependability of the mean RMSD. Distribution analysis found modest negative skewness (cum.3 = -0.329) and moderate positive kurtosis (cum.4 = 0.534), indicating that most conformations clustered near the mean with few deviations. The AtpD-4679 complex involved a longer stabilization period (~5.28 ns), obtaining a plateau RMSD of 0.269 ± 0.023 nm, consistent with minimal conformational modifications following ligand binding. The distribution revealed moderate negative skewness (-0.985) and strong positive kurtosis (2.619), showing tightly concentrated conformations around the mean. In contrast, the AtpD-5029 complex equilibrated faster (~1.26 ns) with a mean RMSD of 0.275 ± 0.027 nm. Slight negative skewness (-0.578) and slight positive kurtosis (0.926) revealed consistent clustering of conformations toward the mean. Overall, RMSD profiles indicated that both ligand-bound systems maintained stable trajectories equivalent to the apo protein, with only slight structural modifications following ligand binding (**Figure 12a**).

Residue-level flexibility across 479 residues of AtpD was assessed using RMSFigure The apo protein exhibited a mean RMSF of 0.116 ± 0.060 nm, with strong positive skewness (cum.3 = 1.416) and mild kurtosis (cum.4 = 2.231), showing that most residues remained solid, while a subset likely located in loop and solvent-exposed regions displayed increased mobility. The AtpD-4679 complex revealed a somewhat higher mean RMSF (0.143 ± 0.071 nm), corresponding with enhanced flexibility in loop regions, terminal ends, and residues near to the binding site (cum.3 = 1.254; cum.4 = 1.903). The AtpD-5029 complex showed a mean RMSF of 0.123 ± 0.066 nm, with flexible residues localized in regions 20-22, 37-38, 106, 125, 155-156, 158, 264, 268-271, 341, 371, 373-392, 414-417, 469, 471-472, and 474-479. These corresponded mostly to loops and solvent-exposed regions, although the protein core remained stiff in all systems. Collectively, RMSF analysis confirmed that ligand interaction did not damage the backbone stability of AtpD but caused localized flexibility in dynamic areas, which may promote conformational adaptation at the binding site (**Figure 12b**). The global compactness of AtpD was examined using Rg analysis. The apo protein had a mean Rg of 2.644 ± 0.013 nm, indicating a stable and compact fold. The AtpD-4679 complex maintained a virtually equal Rg (2.638 ± 0.012 nm), suggesting that pantoprazole binding did not disrupt overall compactness. Similarly, the AtpD-5029 complex revealed a mean Rg of 2.634 ± 0.019 nm, with slight fluctuations in alternate trajectories (1.812-2.361 nm), but without evidence of considerable unfolding. Skewness and kurtosis values across all systems exhibited modest departures from Gaussian distribution, supporting the notion that AtpD preserved a consistent global fold regardless of ligand interaction (**Figure 12c**).

The AtpD-4679 complex displayed an average of 0.26 ± 0.50 H-bonds, showing weak and transitory interactions. Within the 0.35 nm cutoff, however, 1.27 ± 1.21 donor-acceptor pairs were found, suggesting of occasional tight interactions outside rigorous H-bond standards. The higher cumulant values (1.153 and 1.063) revealed strong fluctuations and deviations from Gaussian behavior, compatible with unstable bonding dynamics. The AtpD-5029 complex maintained more stable contacts, with a mean of 0.80 ± 0.65 H-bonds and 1.72 ± 1.31 donor-acceptor pairs within the cutoff. The distribution (cumulants 0.301/0.165 for H-bonds and 0.380/0.029 for pairs) varied only little from Gaussian, consistent with weak but recurring hydrogen bonding (**Figure 12d**).

Collectively, MD simulation analysis reveals that AtpD is structurally stable in both apo and ligand-bound states. Flexibility was mostly restricted to loops and solvent-exposed areas, whereas the protein core remained rigid. Pantoprazole (4679) and rabeprazole (5029) elicited very moderate conformational modifications, with rabeprazole establishing more stable hydrogen bond interactions compared to pantoprazole. Given the key role of AtpD in ATP hydrolysis and energy metabolism of *L. acidophilus*, these findings imply that PPIs can stably interact with AtpD and may potentially influence its functional dynamics.

#### 3.7.4 MD simulation of NadE and PPI bound NadE complexes

**Figure 13:**
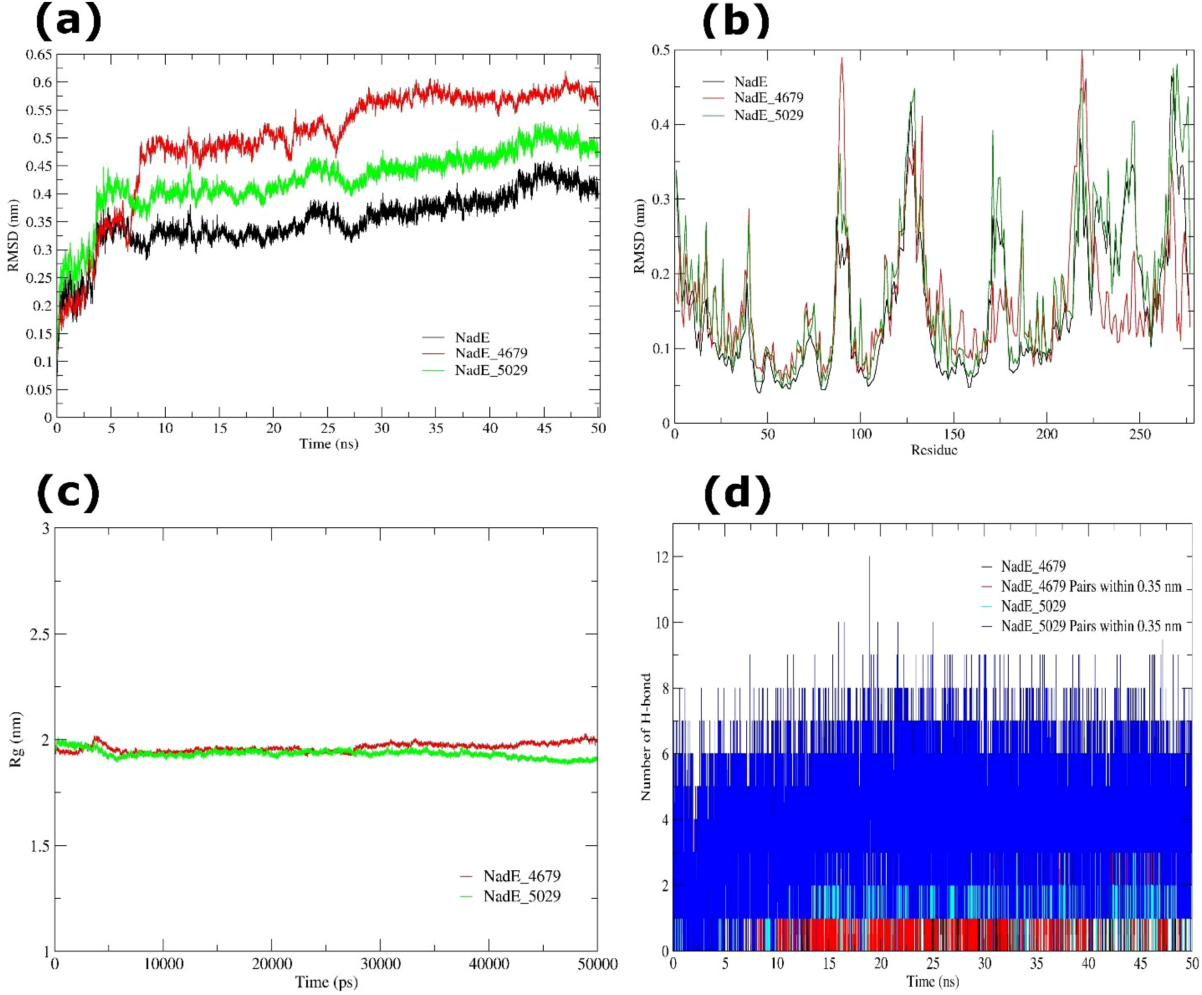
MD simulation of NadE (apo and ligand-bound) systems over 50 ns. **(a)** RMSD trajectories of NadE apo (black), NadE-4679 complex (red), and NadE-5029 complex (blue), indicating structural stability during the simulation. **(b)** RMSF profiles of NadE apo (black), NadE-4679 complex (red), and NadE-5029 complex (green), demonstrating residue-level flexibility and conformational variations. **(c)** Rg analysis of NadE-4679 (red), and NadE-5029 (green) complexes. **(d)** Number of intramolecular H-bond interactions generated by NadE-4679, and NadE-5029 complexes.

The RMSD analysis of the apo NadE protein, estimated over 5001 trajectory frames (dt = 0.01 ns), revealed a mean value of 0.351 ± 0.052 nm, showing general structural stability with relatively minimal variations from the reference conformation. The low SE (3.26 × 10^-4^) validated the trustworthiness of the measurement. Minimal negative skewness (cum.3 = -0.636) and moderate positive kurtosis (cum.4 = 0.790) further showed that most of conformations clustered near the mean with low fluctuations. Upon ligand binding, the NadE-4679 complex exhibited a larger mean RMSD of 0.498 ± 0.103 nm, while the NadE-5029 complex exhibited a comparable value of 0.425 ± 0.057 nm. Both ligand-bound systems displayed mild negative skewness and positive kurtosis, suggesting that binding generated localized conformational modifications without affecting overall structural stability (**Figure 13a**).

Residue-wise RMSF analysis indicated that the apo NadE protein maintained a mean RMSF of 0.151 ± 0.139 nm, with enhanced flexibility predominantly confined to loop regions and solvent-exposed residues. The substantial positive skewness (cum.3 = 2.113) and kurtosis (cum.4 = 4.237) revealed that most residues remained constant, whereas a few exhibited pronounced fluctuations. Ligand binding modestly enhanced flexibility, with mean RMSF values of 0.160 ± 0.084 nm for the NadE-4679 complex and 0.179 ± 0.102 nm for the NadE-5029 complex. Flexible portions were restricted to loops, termini, and surface-exposed areas, whereas the protein core remained stiff (**Figure 13b**). The compactness of the protein was further assessed using Rg analysis. The apo NadE protein demonstrated a mean Rg of 1.972 ± 0.020 nm, which was comparable to values obtained in the NadE-4679 (1.962 ± 0.020 nm) and NadE-5029 (1.933 ± 0.019 nm) complexes. Skewness and kurtosis analysis demonstrated only modest deviations from Gaussian behavior, confirming that ligand attachment did not appreciably alter the global compactness of the protein (**Figure 13c**).

H-bond analysis revealed that the NadE-4679 complex maintained an average of 1.43 ± 0.80 H-bonds throughout the trajectory, based on 1.30 ± 1.06 donor-acceptor pairs within a 0.35 nm cutoff. Low cumulant values suggested near-Gaussian fluctuations compatible with stable H-bond dynamics. The NadE-5029 complex displayed a similar average of 1.37 ± 0.61 H-bonds, but with a substantially higher number of donors-acceptor pairs (4.04 ± 1.67), representing the highest close-contact interactions among the investigated systems. Minor deviations from Gaussian distribution further indicated the persistence of these interactions (**Figure 13d**).

Taken together, the MD simulation analysis finding indicated that NadE retained structurally stable in both apo and ligand-bound states. Pantoprazole (4679) and rabeprazole (5029) produced localized flexibility largely in loops and solvent-exposed areas while preserving the whole structural integrity and compactness of the protein. Given its position as a key enzyme in NAD^+^ production, stable binding of these ligands may affect energy metabolism in *L. acidophilus*.

#### 3.7.5 MD simulation of PyrH and PPI bound PyrH complexes

**Figure 14:**
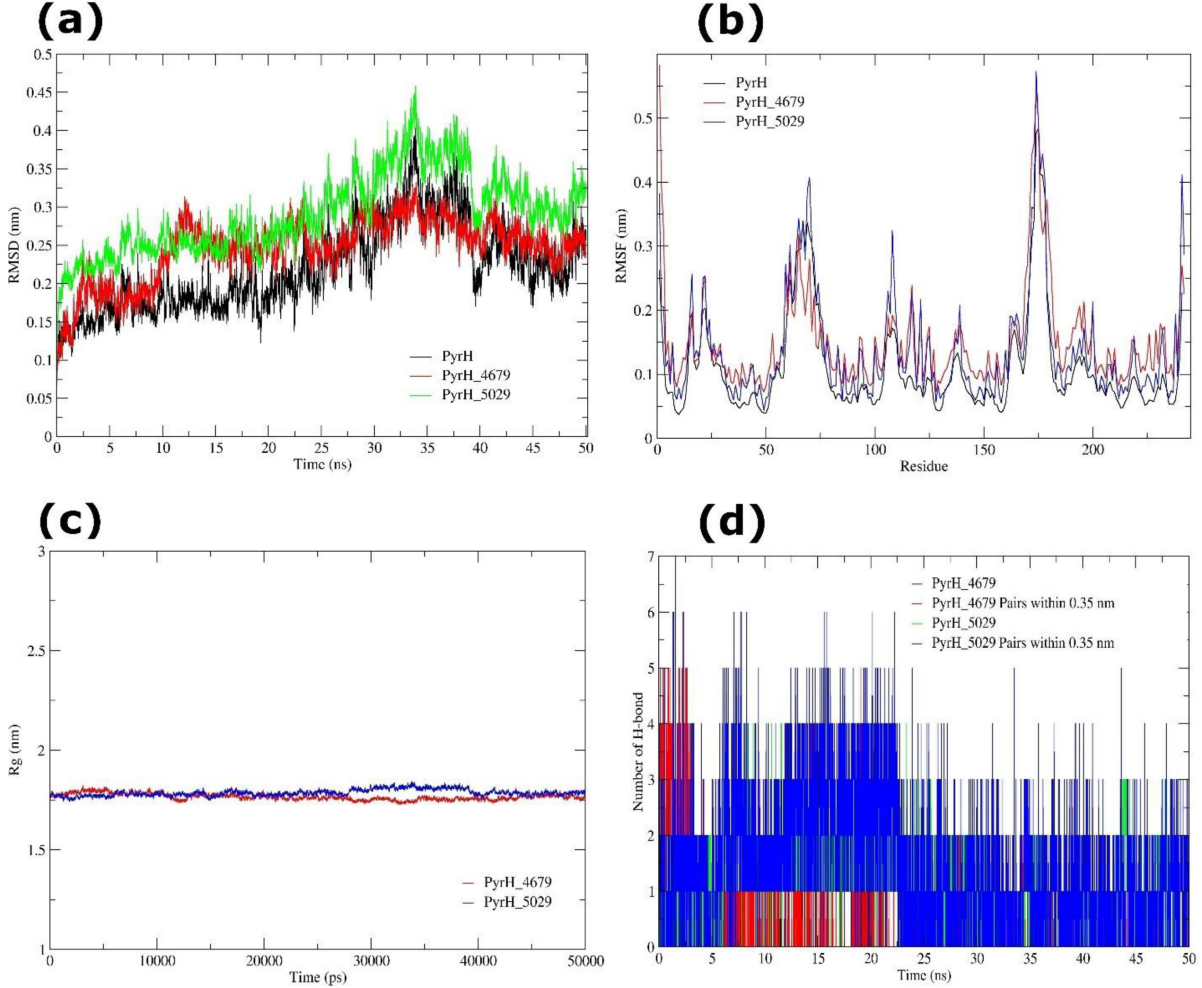
MD simulation of PyrH (apo and ligand-bound) systems over 50 ns.**(a)** RMSD trajectories of PyrH apo (black), PyrH_4679 complex (red), and PyrH_5029 complex (green), indicating structural stability during the simulation. **(b)** RMSF profiles of PyrH apo (black), PyrH_4679 complex (red), and PyrH_5029 complex (green), demonstrating residue-level flexibility and conformational variations. **(c)** Rg analysis of PyrH_4679 (red), and PyrH_5029 (blue) complexes. **(d)** Number of intramolecular H-bond interactions generated by PyrH-4679, and PyrH-5029 complexes.

The RMSD analysis of apo PyrH yielded a mean value of 0.245 ± 0.042 nm, revealing rapid establishment of structural equilibrium with only small fluctuations. Upon ligand binding, distinct conformational responses were identified. The PyrH-4679 complex displayed a much greater RMSD of 0.550 ± 0.290 nm, suggesting extensive conformational rearrangements compatible with an induced-fit mechanism. In contrast, the PyrH-5029 complex retained a moderate RMSD of 0.291 ± 0.053 nm, showing localized changes while keeping overall structural stability (**Figure 14a**).

Residue-level flexibility, measured by RMSF, indicated that the apo protein maintained an average value of 0.110 ± 0.079 nm, with fluctuations predominantly localized to loop and solvent-exposed areas. Pantoprazole binding increased the average RMSF to 0.150 ± 0.078 nm, showing improved flexibility near the binding pocket, whereas rabeprazole binding produced a mild rise to 0.139 ± 0.088 nm, with the catalytic core remaining structurally inflexible (**Figure 14b**). The compactness of the protein was assessed using Rg analysis. The apo PyrH protein revealed an average Rg of 1.972 ± 0.020 nm, which remained comparable upon pantoprazole binding (1.962 ± 0.020 nm). Rabeprazole binding caused a slightly reduced Rg of 1.786 ± 0.017 nm, suggesting the persistence of the global fold with minor ligand-induced compaction (**Figure 14c**).

The PyrH-4679 complex established a mean of 0.34 ± 0.61 H-bonds, showing relatively weak and transitory interactions. Within the 0.35 nm limit, 0.40 ± 0.85 donor-acceptor pairs were detected, suggesting only infrequent close interactions. The comparatively high cumulant values (1.233/1.416 for H-bonds and 1.698/2.647 for pairs) suggested considerable deviations from Gaussian behavior, consistent with unstable hydrogen bonding. Conversely, the PyrH-5029 complex exhibited a mean of 1.04 ± 0.87 H-bonds, with 1.45 ± 1.22 donor-acceptor pairs inside the cutoff, showing weak but recurring stabilizing interactions. The low cumulant values (0.262/-0.168 for H-bonds and 0.501/0.067 for pairs) revealed near-Gaussian behavior, indicating moderately stable hydrogen bonding dynamics (**Figure 14d**).

The MD simulation analysis shows that pantoprazole binding produces considerable conformational rearrangements in PyrH through an induced-fit mechanism, however rabeprazole exhibits overall structural stability with just localized flexibility, revealing unique ways of inhibition. These results suggest that pantoprazole and rabeprazole interact with PyrH could disrupt PyrH-mediated nucleotide metabolism and thus impede the growth of *L. acidophilus*.

#### 3.7.6 MD simulation of Def and PPI bound Def complexes

**Figure 15:**
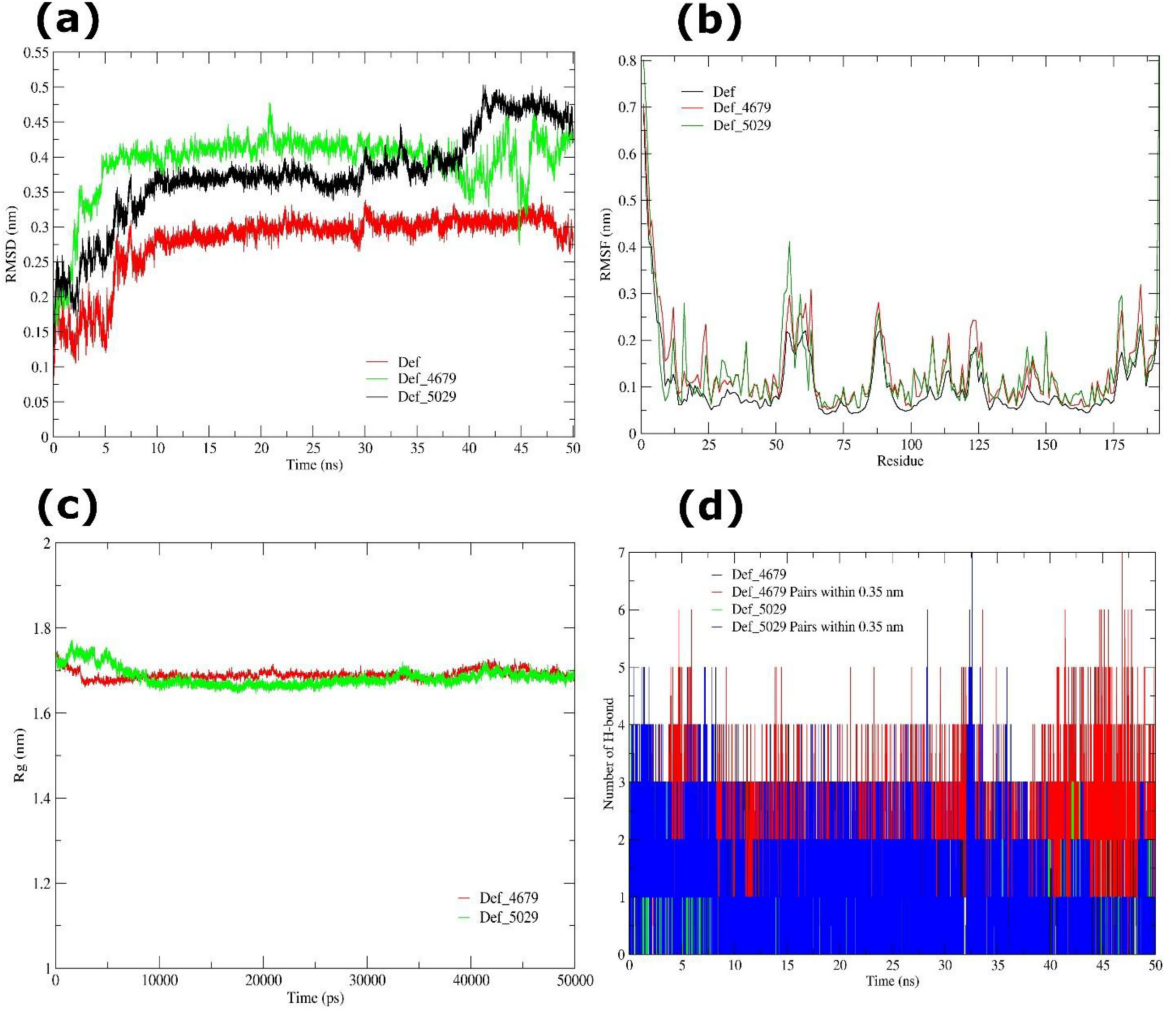
MD simulation of Def (apo and ligand-bound) systems over 50 ns. **(a)** RMSD trajectories of apo Def (red), Def-4679 complex (green), and Def-5029 complex (black), indicating structural stability during the simulation. **(b)** RMSF profiles of apo Def (black), Def-4679 complex (red), and Def-5029 complex (green), illustrating residue-level flexibility and conformational variations. **(c)** Rg analysis of Def-4679 (red), and Def-5029 (green) complexes. **(d)** Number of intramolecular H-bond interactions generated by Def-4679, and Def-5029 complexes.

The RMSD profile of apo Def, analysed throughout 25,001 trajectory frames (dt = 0.002 ns), achieved 0.279 ± 0.047 nm mean, revealing rapid establishment of structural equilibrium with minimal fluctuations over the simulation. The low SE (2.98 × 10^-4^) supported the dependability of the mean value. Distribution analysis found negative skewness (cum.3 = -1.278) and moderate positive kurtosis (cum.4 = 1.066), indicating that most conformations clustered tightly toward the mean with occasional greater deviations. Upon ligand binding, the pantoprazole (4679) complex displayed a slightly larger mean RMSD of 0.393 ± 0.048 nm, with pronounced negative skewness (-1.783) and enhanced kurtosis (3.139), consistent with localized conformational alterations while preserving overall stability. The rabeprazole (5029) complex revealed an RMSD of 0.375 ± 0.066 nm, with reduced skewness (-0.437) and slight positive kurtosis (0.326), showing minimal local flexibility without perturbing the global fold (**Figure 15a**).

Residue-level flexibility, measured via RMSF, suggested that apo Def exhibited 0.102 ± 0.079 nm mean RMSF (SE: 5.70 × 10^-^³), with increased fluctuations confined to loop and solvent-exposed regions (cum.3 = 2.368; cum.4 = 6.691). Pantoprazole binding elevated the mean RMSF to 0.137 ± 0.089 nm (cum.3 = 1.712; cum.4 = 3.727), whereas rabeprazole induced a comparable RMSF of 0.138 ± 0.111 nm (cum.3 = 2.649; cum.4 = 7.681), reflecting ligand-induced flexibility limited to dynamic regions while the structural core remained rigid (**Figure 15b**). Global compactness, measured using Rg, indicated that apo Def kept a compact and stable structure, with an average Rg of 1.700 ± 0.011 nm (SE: 1.55 × 10^-4^). Ligand binding generated only minimal changes in compactness, with pantoprazole- and rabeprazole-bound complexes having mean Rg values of 1.690 ± 0.011 nm and 1.683 ± 0.021 nm, respectively, indicating retention of the global fold along the 50 ns trajectory (**Figure 15c**). Skewness and kurtosis investigations indicated near-Gaussian distributions across all states, representing robust structural stability.

The Def-4679 complex maintained a mean of 1.37 ± 0.75 H-bonds, equating to one to two stable interactions throughout the simulation. Within a 0.35 nm limit, 2.14 ± 1.01 donor-acceptor pairs were detected, reflecting additional close interactions, with distributions consistent with steady dynamics. In contrast, the Def-5029 complex generated fewer H-bonds (0.63 ± 0.67 on average) and donor-acceptor pairs (0.92 ± 0.93), with modest deviations from Gaussian behavior (cumulants 0.554/0.300 for H-bonds and 0.528/0.121 for pairs), suggesting weaker and more transient interactions (**Figure 15d**).

These MD simulations result reveal that pantoprazole and rabeprazole generate only minimal structural flexibility in Def without affecting its global fold. The higher and more persistent hydrogen bonding in the pantoprazole-bound state suggests considerably increased stability, indicating the possibility of these PPIs to reduce Def’s enzymatic activity and consequently disrupt nucleotide synthesis and bacterial growth.

### 3.8 Antimicrobial response of *L. acidophilus* to proton pump inhibitors exposure

**Figure 16:**
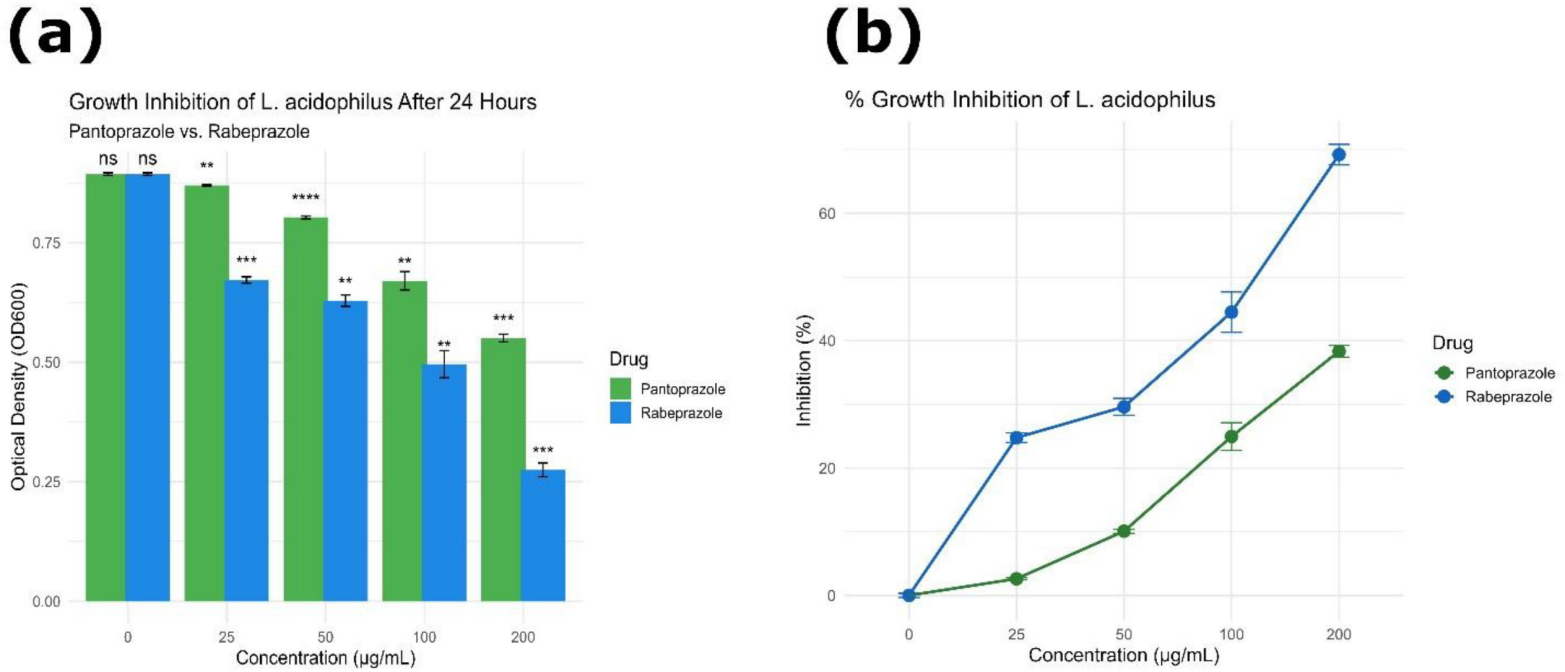
Dose-dependent effects of pantoprazole and rabeprazole on L. acidophilus (ATCC 53544) growth and inhibition. **(a)** Mean OD_600_ ± SD of *L. acidophilus* treated with increasing concentrations of pantoprazole and rabeprazole (0-200 µg/mL). Both drugs reduced growth in a dose-dependent manner, with rabeprazole showing stronger inhibition. **(b)** Percentage (%) growth inhibition (mean ± SEM) relative to untreated controls. Pantoprazole induced mild-to-moderate inhibition, whereas rabeprazole produced higher inhibition across all doses.

The antibacterial effects of pantoprazole and rabeprazole against *L. acidophilus* (ATCC 53544) were examined by assessing growth inhibition across increasing drug concentrations (0-200 µg/mL). Optical density (OD_600_) readings dropped significantly with increasing concentrations for both medications, suggesting a dose-dependent inhibitory effect (**Figure 16**).

Pantoprazole exposure resulted in a gradual decline in bacterial growth, with mean OD values dropping from 0.893 ± 0.005 at 0 µg/mL to 0.551 ± 0.014 at 200 µg/mL (**Figure 16a**). Significant inhibition compared with untreated control was detected starting at 25 µg/mL (p = 0.0064), and extremely significant effects were observed at 50 µg/mL (p = 2.85×10^-5^) and 200 µg/mL (p = 1.41×10^-4^). Correspondingly, percent inhibition rose in a concentration-dependent manner from 2.6% at 25 µg/mL to 38.3% at 200 µg/mL (**Figure 16b**). At 100 µg/mL, inhibition reached 25.0%, showing partial but substantial suppression of *L. acidophilus* growth. Nonlinear dose-response modeling showed the IC^50^ for pantoprazole to be 91.28 ± 9.51 µg/mL, showing considerable inhibitory activity. Rabeprazole had a higher growth-suppressive impact than pantoprazole at equal concentrations. OD values declined rapidly from 0.893 ± 0.005 (control) to 0.672 ± 0.012, 0.629 ± 0.020, and 0.496 ± 0.049 at 25, 50, and 100 µg/mL, respectively. All concentrations exhibited significant differences from control (p < 0.005), with the highest inhibition found at 200 µg/mL (p = 3.40×10^-4^). Growth inhibition increased considerably with dose, reaching 24.8% at 25 µg/mL, 29.6% at 50 µg/mL, 44.5% at 100 µg/mL, and 69.2% at 200 µg/mL. Despite the greater inhibitory pattern, dose-response modelling suggested a substantially larger IC^50^ value of 1103.08 ± 1240.64 µg/mL, suggesting variability and a less consistent sigmoidal response curve compared with pantoprazole. The concentrations used in this study (up to 200 µg/mL) were selected to establish a comprehensive dose-response relationship. While these levels may exceed typical systemic plasma concentrations, localized luminal exposure in the gastrointestinal tract following oral administration may transiently reach higher levels, particularly in microenvironments. Therefore, the findings should be interpreted as representing upper-bound or localized exposure scenarios rather than steady-state physiological concentrations.

Overall, both PPIs displayed dose-dependent inhibition of *L. acidophilus*, but rabeprazole provided stronger % inhibition at all matched dosages, especially at 100 µg/mL and 200 µg/mL. However, pantoprazole produced a more consistent and predictable pharmacodynamic response, reflected in a lower and more reliable IC^50^ estimate. These findings combined show that whereas rabeprazole produces larger initial inhibitory effects, pantoprazole shows more sustained inhibitory kinetics across the investigated dosage range.

### 3.9 qPCR analysis of gene expression changes in *Lactobacillus acidophilus* after PPI exposure

**Figure 17:**
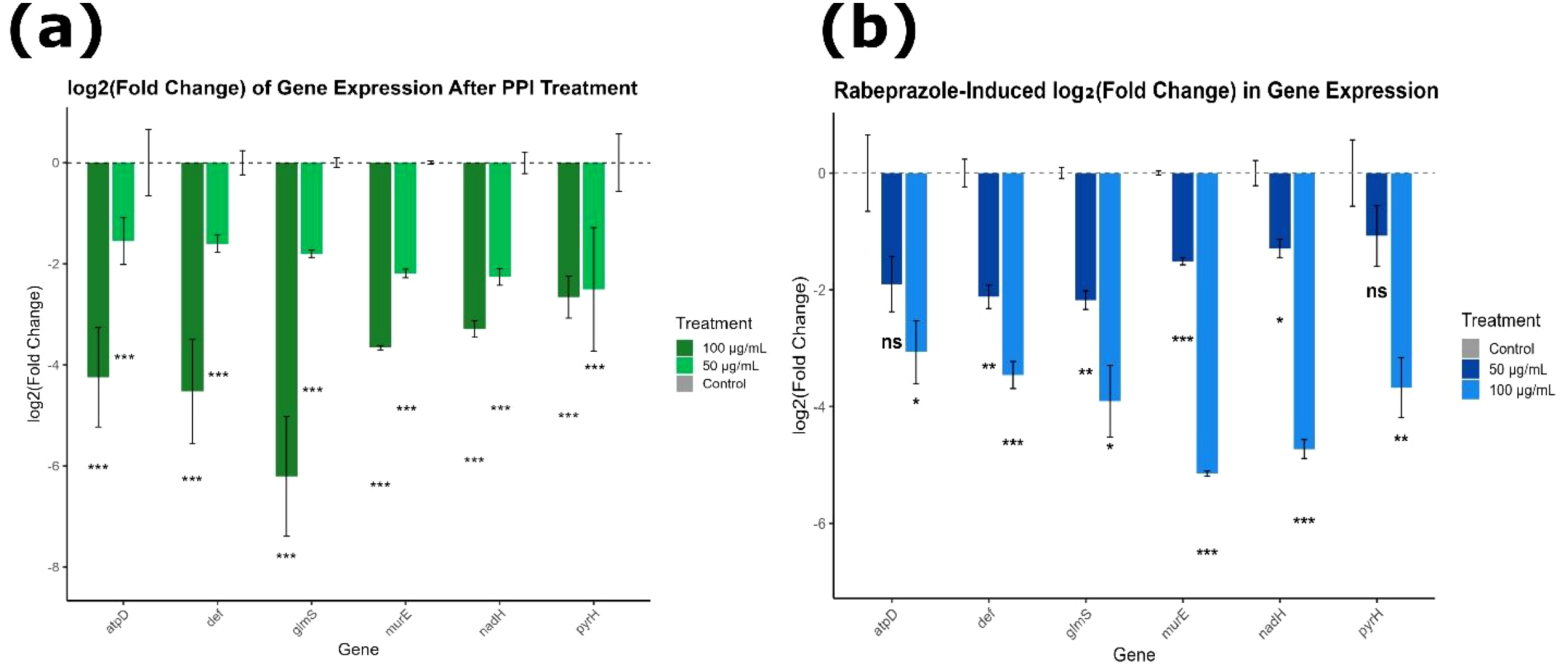
Proton pump inhibitors elicit widespread transcriptional suppression in L. acidophilus. The log_2_(Fold Change) expression of key metabolic and cell-wall related genes following treatment with **(a)** pantoprazole and **(b)** rabeprazole (50 and 100 µg/mL), respectively. Gene expression was normalized to 16S rRNA and estimated using the 2^-^ΔΔCt method. Data reflect mean ± SEM (n = 3). Both PPIs produced dose-dependent increases in ΔΔCt values, leading to lower transcript abundance compared with the untreated control. A dashed line indicates no change relative to control. Statistical significance was assessed using two-tailed t-tests with Benjamini-Hochberg correction. *p< 0.05; **p< 0.01; ***p< 0.001; ns, not significant.

Quantitative real-time (qRT)-PCR analysis normalized to 16S rRNA indicated that both pantoprazole and rabeprazole elicited substantial, dose-dependent suppression of key genes in *L. acidophilus* (ATCC 53544). Pantoprazole induced highly significant suppression across all genes at both 50 µg/mL and 100 µg/mL, with comparable p-values (p = 1.49 × 10^-8^; BH-adjusted p = 1.49 × 10^-8^), highlighting a consistent and substantial inhibitory action (**Figure 17a**).

Pantoprazole significantly lowered the expression of genes involved in energy metabolism. For *atpD*, ΔΔCt rose from 0 in the control to 1.55 and 4.24 at 50 and 100 µg/mL, generating log_2_(Fold Change) values of -1.55 and -4.24. Also, *nadH* showed ΔΔCt values of 2.26 and 3.28, corresponding to log_2_FC values of -2.26 and -3.28, showing significant inhibition of ATP synthesis and NADH-dependent metabolic activity.

Genes involved in cell-wall biosynthesis were even more strongly suppressed. The expression of *murE* increased to ΔΔCt = 2.19 and 3.66 at the two pantoprazole concentrations, while *def* increased to ΔΔCt = 1.61 and 4.52; both genes showed highly significant downregulation. The most profound repression occurred in *glmS*, where ΔΔCt values increased to 1.80 and 6.21 at 50 and 100 µg/mL, corresponding to dramatic log_2_FC reductions of -1.80 and -6.21. Even the essential gene *pyrH* was significantly inhibited, with ΔΔCt values of 2.51 and 2.66 (log_2_FC-2.51 and -2.66. These results confirm that pantoprazole exerts uniform and highly significant transcriptional inhibition across multiple metabolic and structural pathways.

Rabeprazole elicited a similarly broad but more variable pattern of transcriptional repression (**Figure 17b**). Across all genes, rabeprazole significantly increased ΔCt and ΔΔCt values and reduced fold change expression relative to the control, but the statistical strength varied according to gene and dose. For *atpD*, ΔΔCt reached 3.07 at 50 µg/mL and 1.90 at 100 µg/mL, producing log_2_FC values of -3.07 and -1.90. The 50µg/mL rabeprazole treatment showed significant suppression (*p* = 0.0086; *p*_adj = 0.0103), whereas the 100 µg/mL rabeprazole treated condition did not reach significance after adjustment (*p* = 0.0498; *p*_adj = 0.0598; ns). Rabeprazole significantly repressed *def*, with ΔΔCt values of 3.46 and 2.12 corresponding to log_2_FC values of -3.46 and -2.12. Both treatments were statistically significant (treated with 50 µg/mL rabeprazole: *p*_adj = 0.0022; 100µg/mL rabeprazole: *p*_adj = 0.0022). Strong inhibition was also observed for *glmS*, which showed ΔΔCt values of 3.91 and 2.18 (log_2_FC-3.91 and -2.18), with both doses significant after adjustment (treated with 50 µg/mL rabeprazole: *p*_adj = 0.0022; 100µg/mL rabeprazole: *p*_adj = 0.0268).

The most pronounced rabeprazole-mediated suppression occurred for *murE*, where ΔΔCt reached 1.51 at 50 µg/mL and 5.15 at 100 µg/mL, corresponding to log_2_FC values of -1.51 and -5.15. Both treatments exhibited extremely strong significance (50µg/mL rabeprazole: p = 2.53 × 10^-7^; *p*_adj = 0.000917; 100 µg/mL rabeprazole: p = 2.53 × 10^-7^; *p*_adj = 0.00163). *nadH* also showed clear, dose-dependent repression, with ΔΔCt values of 1.29 and 4.73, producing log_2_FC values of -1.29 and -4.73. Both doses were highly significant (50µg/mL rabeprazole: *p*_adj = 0.0115; 100µg/mL rabeprazole: *p*_adj = 0.0124). Although *pyrH* displayed increased ΔΔCt values at both concentrations (1.08 and 3.68; log_2_FC-1.08 and -3.68), the variation across replicates prevented statistical significance (*p*_adj = 0.109 for both).

Overall, both pantoprazole and rabeprazole markedly suppressed gene expression in *L. acidophilus*, targeting pathways essential for ATP generation, NADH metabolism, protein maturation, and cell-wall biosynthesis. Pantoprazole exhibited a uniform and highly significant dose-dependent repression across all genes evaluated, showing a broad and consistent inhibitory action on bacterial transcription. In comparison, rabeprazole elicited strong but more gene-specific responses, with the most pronounced suppression observed for *murE, glmS, nadH*, and *def.* The combined ΔΔCt, fold-change, and statistical profiles suggest that while both PPIs compromise critical metabolic and structural pathways essential to bacterial viability, the extent of transcriptional inhibition is generally greater at 100 µg/mL, with pantoprazole demonstrating the most uniform and potent overall effect. Although the antimicrobial assays demonstrated dose-dependent growth inhibition and qRT-PCR analysis showed transcriptional downregulation of selected genes, mRNA-level changes do not necessarily translate into reduced protein activity. In the absence of complementary functional assays such as enzyme activity measurements, ATP/NAD^+^ quantification, or cell wall integrity analyses the observed transcriptional alterations may reflect a generalized stress response rather than specific inhibition of the predicted molecular targets identified through docking. Nevertheless, the consistency between *in silico* predictions and transcriptional trends suggests that the identified pathways may represent plausible targets, warranting further functional validation.

## 4 Discussion

Our findings provide comprehensive molecular and functional evidence on how proton pump inhibitors (PPIs) interact with *Lactobacillus acidophilus*, a key probiotic species essential for metabolic balance and gut microbial stability (Tang et al., 2023), and immunomodulation (Yu et al., 2023). By integrating molecular docking, molecular dynamics (MD) simulations, MIC assays, and transcriptional profiling, it was revealed that PPIs-particularly pantoprazole and rabeprazole could directly disrupt essential bacterial pathways, potentially contributing to PPI-associated gut dysbiosis.

PPIs are acid-activated prodrugs that that transform into reactive sulfenamide intermediates in highly acidic (gastric) environments (Sachs et al., 2006; Shin and Sachs, 2008). It is an important pharmacological factor to take into account when interpreting the present findings. Under the mild acidic culture conditions (pH 5.5) employed in this study, classical acid-mediated activation of PPIs is unlikely to occur. Nevertheless, PPIs are known to exhibit chemical instability in aqueous environments and may undergo partial transformation or generate reactive intermediates even outside strongly acidic conditions (Shin et al., 2009; Wołowiec et al., 2025). Furthermore, experimental studies have shown that PPIs such as omeprazole and lansoprazole can reduce urease activity and bacterial proliferation in *Helicobacter pylori*, suggesting that these drugs can engage with microbial enzymes independently of gastric acid suppression (Nagata et al., 1993; McGowan et al., 1994; Sjöström et al., 1996). Additionally, large-scale microbiome studies have reported significant alterations in gut microbial composition among individuals receiving long-term PPI therapy, suggesting that these drugs may influence microbial metabolic processes and community structure beyond their effects on gastric acidity (Freedberg et al., 2014; Imhann et al., 2016). Therefore, the biological effects observed in the present study may reflect interactions of the parent compounds or partially transformed derivatives with bacterial proteins or metabolic pathways rather than the canonical gastric proton pump inhibition mechanism. These findings support the possibility that PPIs may exert previously underappreciated off-target effects on beneficial members of the gut microbiota.

Docking studies demonstrated that all tested PPIs (pantoprazole, rabeprazole, omeprazole, and lansoprazole) engage with high affinity across multiple essential bacterial proteins specially, Mur protein family, GlmS, NadE, Def (PDF), and PyrH. These enzymes regulate peptidoglycan synthesis (Hervin et al., 2023), protein maturation (Chen et al., 2004), NAD^+^ biosynthesis (Covarrubias et al., 2021), and pyrimidine metabolism (Lee et al., 2007). Pantoprazole showed the strongest and most consistent binding (lowest MolDock and Rerank scores) across MurB, NadE, and PyrH, suggesting broad interference with structural and metabolic pathways. Rabeprazole exhibited the strongest binding to Def, supported by multiple H-bonds with Leu14, Arg56, and Gly112, indicating potential inhibition of post-translational processing (Cai et al., 2006; Gao et al., 2016). The strong binding affinities observed align with prior reports highlighting MurA/MurB (Naorem et al., 2022; Raina et al., 2022; Frlan et al., 2023; Kumar et al., 2023) and NadE (Kim et al., 2013; Rodionova et al., 2014) as attractive antibacterial targets. Our docking analysis showed that several residues interacting with pantoprazole and rabeprazole are located within or adjacent to predicted catalytic or nucleotide-binding regions of the target enzymes. A limitation of this study is that the docking protocol was not benchmarked using experimentally validated inhibitors for several of the selected enzymes, as well-characterized inhibitors specific to *L. acidophilus* proteins such as MurA, MurB, and GlmS are limited. Therefore, the docking results should be interpreted as predictive estimates of ligand-protein interactions rather than definitive evidence of enzymatic inhibition. To strengthen the reliability of these predictions, molecular dynamics simulations were performed to assess the stability of ligand-protein complexes, and qRT-PCR analysis was conducted to evaluate transcriptional responses of selected target genes following PPI exposure.

MD simulations further supported the docking predictions by demonstrating stable protein-ligand interactions throughout the simulation trajectories. The pantoprazole-NadE and pantoprazole-MurB complexes exhibited relatively stable RMSD profiles, indicating minimal structural deviation from the initial docked conformations during the simulation period. Residue-level flexibility assessed by RMSF analysis remained largely confined to peripheral loop regions, while residues within the binding pockets showed limited fluctuations, suggesting stable ligand binding. In addition, the radius of gyration remained relatively constant, indicating that the overall compactness of the protein structures was maintained during the simulation. Persistent H-bond interactions between the ligands and key active-site residues further supported the stability of the complexes. Similarly, the rabeprazole-Def complex maintained stable structural parameters, suggesting that rabeprazole could interfere with peptide deformylation processes. Additional computational analyses, such as electrostatic surface potential mapping, ligand efficiency evaluation, and binding free energy calculations (e.g., MM-PBSA or MM-GBSA), could provide deeper insights into the energetic basis of ligand binding. Essential dynamics approaches, including principal component analysis (PCA) and free energy landscape (FEL) analysis, may further characterize conformational stability of the protein-ligand complexes. Although these analyses were beyond the scope of the present study, they represent valuable directions for future investigations to refine the mechanistic understanding of PPI-protein interactions. The MD simulations performed over a 50 ns timeframe were sufficient to evaluate the stability of the protein-ligand complexes, as supported by consistent RMSD, RMSF, radius of gyration, and hydrogen bond profiles. Nevertheless, longer simulations or replicate runs could provide additional insights into long-term conformational dynamics and further strengthen the robustness of the MD results. Overall, these findings suggest that PPIs form stable interactions with essential bacterial proteins involved in cell-wall precursor synthesis, protein maturation, and nucleotide metabolism.

Functional validation using MIC assays indicated that both pantoprazole and rabeprazole suppress *L. acidophilus* growth in a dose-dependent manner. Pantoprazole demonstrated a smooth, predictable inhibition curve with a moderate IC^50^ of 91.28 ± 9.51 µg/mL, consistent with steady pharmacodynamic behavior. Rabeprazole exerted a stronger immediate inhibitory effect at matched concentrations but produced a variable dose-response pattern with a markedly higher and unstable IC^50^ (1103.08 ± 1240.64 µg/mL). These differences highlight that although rabeprazole may impair growth more sharply at specific concentrations, pantoprazole exerts more reliable and consistent antimicrobial pressure. While PPI-associated dysbiosis is a community-level phenomenon, isolating direct drug-bacterium interactions is a necessary first step. By decoupling host and interspecies effects, this study provides foundational mechanistic insight into how PPIs can directly impair probiotic physiology. Notably, variability observed in the IC^50^ values, particularly for rabeprazole, may reflect experimental variations, compound instability under mild-acidic conditions, or differential response dynamics. Consequently, the dos-response relationships should be interpreted with caution, and the IC^50^ estimates are considered indicative rather than definitive measures of drug potency.

Gene expression profiling corroborated the docking, MD, and MIC results. The selected genes (*murE, glmS, atpD, nadH, def*, and *pyrH*) represent bottleneck enzymes in peptidoglycan synthesis, energy production, NAD^+^ metabolism, and protein maturation. Transcriptional repression of these genes therefore reflects disruption of non-redundant pathways essential for bacterial viability. Pantoprazole induced uniform and highly significant transcriptional repression across all genes examined, with strong increases in ΔCt and ΔΔCt values and pronounced reductions in 2^-^ΔΔCt and log_2_FC at both 50 and 100 µg/mL. Genes involved in ATP generation (*atpD*), NADH metabolism (*nadH*), peptidoglycan synthesis (*murE*, *glmS*), and protein maturation (*def*) were among the most strongly affected.

Rabeprazole also downregulated key genes but exhibited more gene-specific patterns. The strongest effects were observed for *murE*, *glmS*, *def*, and *nadH*, consistent with the binding and MD results. Although *pyrH* and *atpD* were downregulated, their responses showed greater variability, corresponding with rabeprazole’s inconsistent MIC-derived IC^50^ estimates. Although protein abundance and metabolite measurements provide complementary information, transcriptional regulation represents the primary and earliest level of bacterial response to chemical stress. Previous studies have demonstrated strong concordance between transcriptional repression of *mur*, *glm*, *atp*, and *nad* genes and downstream impairment of cell wall synthesis and energy metabolism in Gram-positive bacteria. In this context, targeted qRT-PCR serves as a sensitive and pathway-specific readout of functional perturbation.

Notably, genes encoding proteins with the highest docking stability and MD persistence (MurE, GlmS, NadH, AtpD) also exhibited the most pronounced and consistent transcriptional repression, reinforcing a causal link between predicted binding and functional gene regulation. Collectively, the docking and MD analyses identify high-affinity, stable interactions between PPIs and essential metabolic and structural proteins of *L. acidophilus*. MIC assays confirm that these molecular interactions translate into functional growth inhibition. qPCR analysis further determines that PPIs suppress transcription of the precise pathways predicted by docking and MD to be targeted including cell-wall biosynthesis, NAD^+^ metabolism, and energy production.

The observed dose-dependent growth regulation and transcriptional suppression indicate that biologically active species were present during the experimental exposure period, despite the absence of a protonation (acid-activation) step. This supports the possibility of non-canonical interactions between PPIs (or their intermediate fractions) and house-keeping bacterial proteins. Overall, our findings align with clinical reports linking PPI therapy to gut dysbiosis, decreased microbial diversity, and increased susceptibility to infections (Drall et al., 2018; Naito et al., 2018; Zhang et al., 2023; Fossmark and Olaisen, 2024). Long-term PPI use has also been associated with increased mortality risk (Bruno et al., 2019; Maideen, 2023), likely mediated at least in part by microbiome disruption. Our results provide mechanistic evidence supporting these clinical observations by showing that PPIs directly impair probiotic viability and function through inhibition of essential bacterial enzymes and pathways.

This study has several limitations that should be considered when interpreting the findings. First, the experimental design is based on *in vitro* conditions using a single bacterial species, *Lactobacillus acidophilus*, which does not fully capture the complexity and diversity of the human gut microbiome. *In vivo*, microbial communities exist within a dynamic and interactive ecosystem involving multiple bacterial taxa, host factors, and environmental influences, all of which can modulate drug-microbe interactions. Second, the controlled laboratory conditions employed in this study do not replicate the physicochemical heterogeneity of GIT, including variations in pH, nutrient availability, and microbial crosstalk. As a result, the observed growth inhibition and transcriptional responses may differ in a multi-species or host-associated environment. Third, while transcriptional analysis provides insights into gene regulation, the absence of complementary functional assays limits the ability to directly link these changes to protein activity or metabolic outcomes. Additionally, the concentrations tested represent upper-range exposure conditions and may not fully reflect physiological intestinal levels of active PPIs. Collectively, these limitations suggest that the findings should be interpreted as preliminary and species-specific, providing a mechanistic basis that requires further validation in more complex *in vitro* systems, multi-species models, and *in vivo* studies.

These findings suggest that widely used non-antibiotic drugs such as PPIs may exert previously underrecognized direct molecular effects on commensal bacteria. Such interactions may contribute to microbiome alterations reported in long-term PPI users and highlight the importance of considering drug–microbe interactions in microbiome-related health outcomes.

## 5 Conclusion

This study reveals that PPIs exert significant off-target effects on *Lactobacillus acidophilus* by binding to and potentially inhibiting essential enzymes involved in cell-wall biosynthesis, nucleotide and amino-sugar metabolism, protein maturation, and energy production. Molecular docking and MD simulations identified highly stable PPI-protein interactions, which were corroborated experimentally by dose-dependent growth inhibition and widespread transcriptional repression of key metabolic genes. Among the tested PPIs, pantoprazole demonstrated the most consistent inhibitory effect, whereas rabeprazole produced stronger but more variable suppression. These findings provide mechanistic evidence supporting clinical observations of PPI-associated gut dysbiosis and reduced *Lactobacillus* abundance. A hypothesis-based selection of essential genes predicted computationally was employed, allowing precise mechanistic analysis and limiting noise from unrelated or compensatory effects, instead of performing untargeted transcriptomics. The findings also underscore the importance of evaluating off-target effects of widely prescribed medications on commensal microbes. This study focused on direct molecular and transcriptional effects of PPIs on *L. acidophilus* using integrative computational modeling and targeted gene expression analysis. While protein-level and metabolite measurements were beyond the scope of this work, the strong concordance between docking, molecular dynamics stability, and dose-dependent transcriptional repression of essential metabolic genes supports the proposed mechanism. Future studies may extend these findings to broader microbial communities and host-associated conditions. The human gut microbiota comprises a highly diverse and complex community of microorganisms. Therefore, the responses observed in this study with *L. acidophilus* as a model probiotic bacteria may not be directly extrapolated to other bacterial taxa. Therefore, further investigations involving multiple species and community-level models will be necessary to determine the broader applicability of these findings.

## Data Availability

The original contributions presented in the study are included in the article/**Supplementary Material**. Further inquiries can be directed to the corresponding author.
