# Supplementary material for: Proton pump inhibitor exposure modulates functional and transcriptional responses in Lactobacillus acidophilus: a comprehensive computational and experimental insights
Source: Front Cell Infect Microbiol. 2026 May 8;16:1781831. doi: 10.3389/fcimb.2026.1781831 (PMC13194612; doi:10.3389/fcimb.2026.1781831)
Supplement: Supplementary file 1 [file Table1.docx]

Supplementary Material

# Supplementary Data

# Supplementary Figures and Tables

## Supplementary Figures


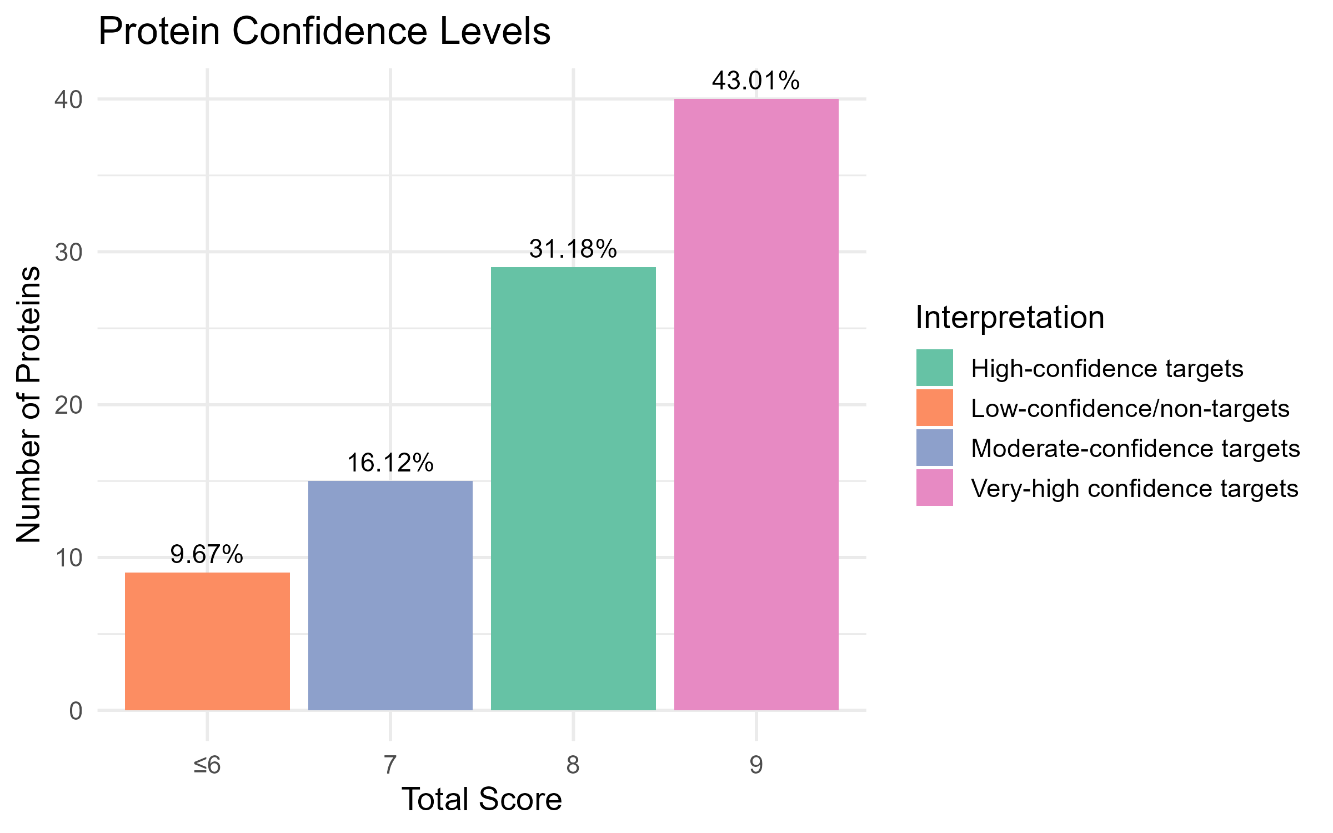


**Supplementary Figure 1.** Distribution of proteins by total score, colored by confidence level: very-high, high, moderate, and low/non-targets.


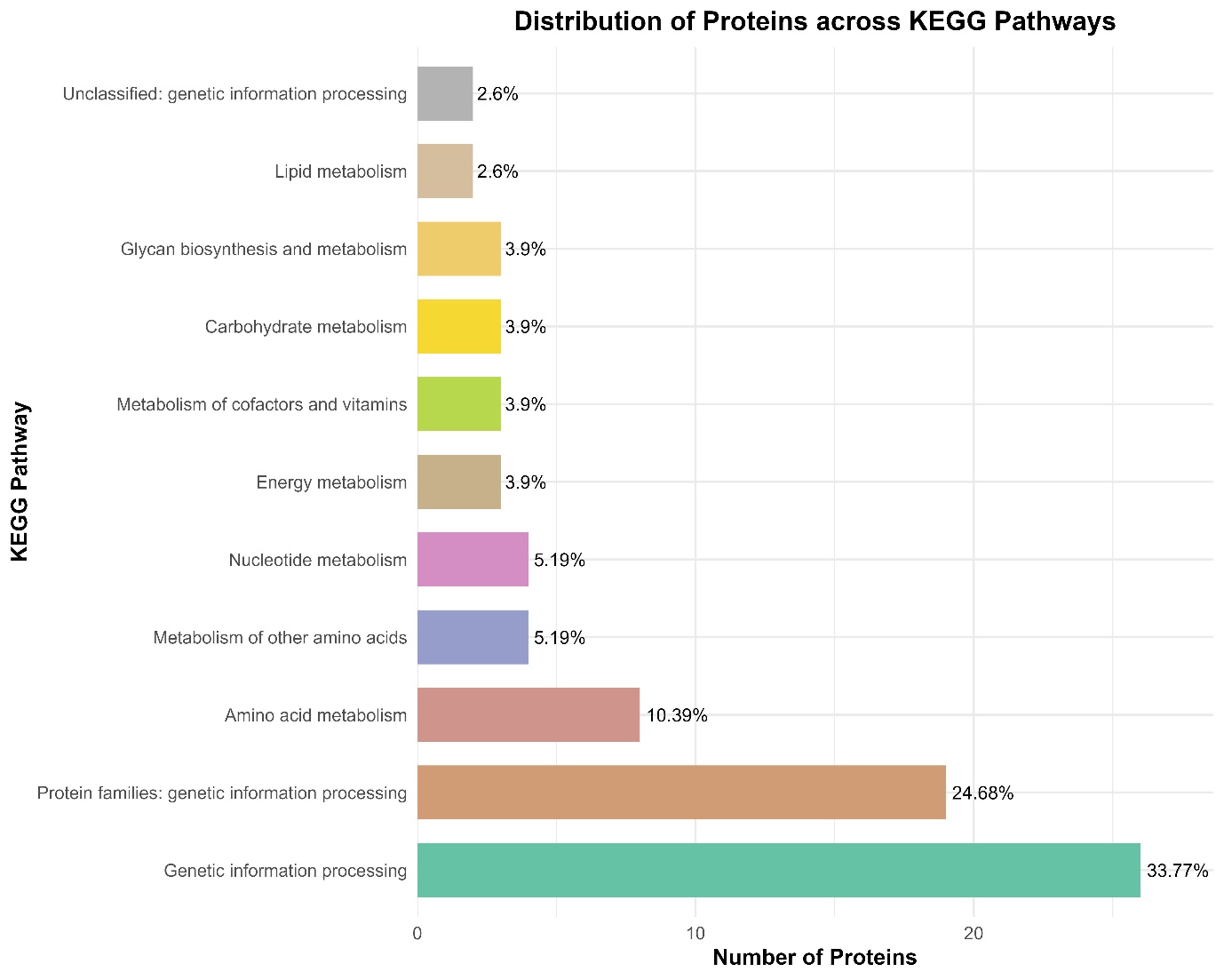


**Supplementary Figure 2.** Bar plot demonstrating the functional categorization of identified proteins into KEGG pathways

**Supplementary Table 1.** The primer sets used for qPCR expression study

| **Sl. No.** | **Gene** | **Primers** | **Seq (5’-3’)** |
| --- | --- | --- | --- |
| 1. | *nadH* | NadH-F | GGTTGCCCTAAGCACCTTTA |
|  |  | NadH-R | GTTACACCCAGAGCTACTTCATC |
| 2. | *pyrH* | PyrH-F | GGGCGTTCCGACTAGAATG |
|  |  | PyrH-R | CAGTACCACCACCGAAGATTAC |
| 3. | *atpD* | AtpD-F | GGTCCAGTATCAGACATGATGG |
|  |  | AtpD-R | GCTCATTAGCAAGCGTTTCAG |
| 4. | *def* | Def-F | ATGCAAGTGGCAGTTGATTTAC |
|  |  | Def-R | CTCCAATCATATTGGCTGCTAAAC |
| 5. | *glmS* | GlmS-F | CGCTGGACTTGTTGGTAAGA |
|  |  | GlmS-R | GCTGTTTCACCTGATTGAGTTAAG |
| 6. | *murE* | MurE-F | GGGAAAGACTACTTCGGCATAC |
|  |  | MurE-R | TTGGCCCAACAACATCATTAAC |

**Supplementary Table 2.** Subtractive genomic approach for identifying essential non-homologous proteins in *L. acidophilus* core proteome

| **Step of Filtering Pipeline** | **Number of Proteins Retained** |
| --- | --- |
| Core Proteome (non-redundant) | 1532 |
| Essential proteins (DEG) | 279 |
| Subcellular localization | 233 |
| Non-homologous proteins | 93 |

**Supplementary Table 3.** Identification of essential metabolic and biosynthetic functions through KEGG pathway analysis

| **KEGG Pathway** | **KEGG ID** | **Gene Symbol** | **Protein Name** | **Functional Role** |
| --- | --- | --- | --- | --- |
| **Glycan biosynthesis & metabolism** | K00075 | *murB* | UDP-N-acetylmuramate dehydrogenase | Catalyzes reduction step in peptidoglycan precursor biosynthesis |
| 00520 Amino sugar & nucleotide sugar metabolism | K00790 | *murA* | UDP-N-acetylglucosamine 1-carboxyvinyltransferase | First committed step in peptidoglycan biosynthesis |
|  | K00820 | *glmS* | Glutamine-fructose-6-phosphate transaminase (isomerizing) | Provides UDP-GlcNAc precursors for glycan metabolism |
|  | K00963 | *galU* | UTP-glucose-1-phosphate uridylyltransferase | Generates UDP-glucose for nucleotide sugar metabolism |
| 00541 Nucleotide sugar biosynthesis | K00963 | *galU* | UTP-glucose-1-phosphate uridylyltransferase | UDP-glucose formation for nucleotide sugar pathways |
| 00550 Peptidoglycan biosynthesis | K01921 | *ddl* | D-alanine-D-alanine ligase | Produces D-Ala-D-Ala dipeptide for cell wall crosslinking |
|  | K01925 | *murC* | UDP-N-acetylmuramoyl-L-alanine-D-glutamate ligase | Adds D-glutamate to peptidoglycan precursors |
|  | K01928 | *murE* | UDP-N-acetylmuramoyl-L-alanyl-D-glutamate ligase | Incorporates meso-diaminopimelate into peptidoglycan precursors |
|  | K01929 | *murF* | UDP-N-acetylmuramoyl-tripeptide ligase | Final cytoplasmic step in peptidoglycan precursor synthesis |
|  | K07009 | *cobS* | Cobyric acid synthase | Cobalamin biosynthesis, cross-linked with peptidoglycan-related annotations |
| **Nucleotide metabolism** | K00526 | *nrdB* | Ribonucleotide-diphosphate reductase subunit beta | Converts ribonucleotides to deoxyribonucleotides |
| 00240 Pyrimidine metabolism | K00945 | *cmk* | (d)CMP kinase | Catalyzes phosphorylation of CMP to CDP |
|  | K01520 | *dut* | dUTP diphosphatase | Hydrolyzes dUTP to dUMP, preventing uracil misincorporation |
|  | K09903 | *pyrH* | UMP kinase | Converts UMP to UDP in pyrimidine metabolism |
| **Amino acid metabolism** | K00820 | *glmS* | Glutamine-fructose-6-phosphate transaminase | Alanine, aspartate & glutamate metabolism |
| 00260 Gly/Ser/Thr metabolism | K00928 | *ask* | Aspartate kinase | Initiates biosynthesis of lysine, threonine, methionine |
| 00270 Cys/Met metabolism | K00928 | *ask* | Aspartate kinase | Common entry enzyme for cysteine & methionine biosynthesis |
| 00300 Lysine biosynthesis | K00215 | *dapB* | 4-hydroxy-tetrahydrodipicolinate reductase | Lysine pathway intermediate reduction |
|  | K01714 | *dapA* | 4-hydroxy-tetrahydrodipicolinate synthase | Catalyzes an early step in lysine biosynthesis |
|  | K01778 | *dapF* | Diaminopimelate epimerase | Converts LL-DAP to meso-DAP for peptidoglycan & lysine synthesis |
|  | K05822 | *dapL* | 2,3,4,5-tetrahydropyridine-2,6-dicarboxylate N-acetyltransferase | Alternative lysine biosynthesis route |
| **Energy metabolism** | K02111 | *atpA* | F0F1 ATP synthase subunit alpha | ATP generation in oxidative phosphorylation & photosynthesis |
| 00190 Oxidative phosphorylation | K02112 | *atpD* | F0F1 ATP synthase subunit beta | Catalytic site for ATP synthesis |
|  | K02114 | *atpC* | F0F1 ATP synthase subunit epsilon | Regulatory subunit of ATP synthase |
| Carbon fixation (00710) | K01624 | *fbaA* | Class II fructose-1,6-bisphosphate aldolase | Calvin cycle, carbon fixation |
| Carbon fixation (00720) | K00625 | *pta* | Phosphate acetyltransferase | Generates acetyl-CoA, links carbon fixation & energy metabolism |

**Supplementary 4**. Evaluation of GalaxyRefine-processed 3D structural models of prioritized target proteins

| **3D Refine Protein** | **ERRAT** | **VERIFY3D** | **Ramachandran’s favor regions (%)** | **Z-Score (ProSA)** |
| --- | --- | --- | --- | --- |
| MurA | 99.0244 | 71.66 | 92.8 | -11.86 |
| MurB | 98.25781 | 86.58 | 97,2 | -9.36 |
| MurE | 97.8474 | 83.75 | 95.5 | -11.01 |
| MurF | 97.7629 | 93.63 | 95.8 | -11.59 |
| GlmS | 95.755 | 82.92 | 95.9 | -12.23 |
| GalU | 96.9072 | 72.00 | 96.1 | -10.04 |
| GatD | 98.6301 | 99.56 | 94.9 | -8.03 |
| DapA | 98.0132 | 80.39 | 94.1 | -9.48 |
| DapF | 96.9231 | 96.11 | 95.3 | -9.55 |
| Def | 95.9538 | 73.82 | 95.8 | -6.53 |
| NadE | 96.6292 | 64.49 | 97.1 | -7.27 |
| PyrH | 99.569 | 71.78 | 97.5 | -8.53 |
| AtpA | 98.75 | 79.72 | 96.5 | -10.85 |
| AtpC | 95.6522 | 58.90 | 98.5 | -6.05 |
| AtpD | 98.2942 | 80.38 | 96.3 | -11.36 |

**Supplementary 5**. Assessment of druggability of 3D refined target proteins using FPocket

| **3D Refine Protein** | **Score** | **Druggability** | **Alpha Sphere** | **Volume** |
| --- | --- | --- | --- | --- |
| MurA | 1.074 | 0.996 | 137 | 824.305 |
| MurB | 1.318 | 1.000 | 142 | 1236.233 |
| MurE | 1.447 | 0.997 | 146 | 1228.327 |
| MurF | 1.031 | 0.978 | 89 | 751.753 |
| GlmS | 1.598 | 0.996 | 106 | 1422.930 |
| GalU | 1.151 | 0.981 | 124 | 1074.291 |
| GatD | 0.849 | 0.899 | 39 | 435.276 |
| DapA | 0.803 | 0.481 | 29 | 572982 |
| DapF | 0.832 | 0.867 | 50 | 659.019 |
| Def | 0.783 | 0.866 | 45 | 425.959 |
| NadE | 1.467 | 1.00 | 165 | 1580.323 |
| PyrH | 0.987 | 0.967 | 56 | 870.107 |
| AtpA | 0.932 | 0.908 | 78 | 564.577 |
| AtpC | 0.707 | 0.754 | 30 | 349.314 |
| AtpD | 1.091 | 0.975 | 65 | 937.374 |

**Supplementary Table 6:** Molecular docking simulation of essential proteins with proton pump inhibitors (PPIs)

| **Protein** | **Pose** | **Ligand** | **MolDock Score** | **Rerank Score** | **HBond**  **Energies** |
| --- | --- | --- | --- | --- | --- |
| MurA | [00] 3883 | 3883 | -119.37 | -90.061 | -2.5 |
| MurA | [01] 4594 | 4594 | -110.23 | -93.639 | -8.5233 |
| **MurA** | **[01] 4679** | **4679** | **-124.85** | **-95.314** | **-11.087** |
| **MurA** | **[00] 5029** | **5029** | **-126.91** | **-91.749** | **-5.388** |
| **MurA** | **[00] 9568614** | **9568614** | **-125.57** | **-100.17** | **-3.9649** |
| MurA | [04] 9568614 | 9568614 | -119.58 | -97.076 | -8.9904 |
| MurA | [00] 9578005 | 9578005 | -124.29 | -99.272 | -9.6284 |
| MurB | [01] 3883 | 3883 | -136.43 | -112.58 | 0 |
| **MurB** | **[01] 4594** | **4594** | **-132.29** | **-70.053** | **-6.4281** |
| MurB | [00] 4679 | 4679 | -127.96 | -105.26 | -9.316 |
| **MurB** | **[01] 5029** | **5029** | **-153.87** | **-120.43** | **-5.9913** |
| MurB | [00] 9568614 | 9568614 | -129.52 | -94.595 | -7.5185 |
| MurB | [01] 9578005 | 9578005 | -124.29 | -96.68 | -2.7276 |
| MurE | [00] 3883 | 3883 | -144.63 | -120.06 | -2.26 |
| **MurE** | **[02] 4679** | **4679** | **-153.15** | **-126.68** | **-3.569** |
| **MurE** | **[00] 9568614** | **9568614** | **-150.3** | **-126.26** | **-3.1265** |
| MurE | [00] 4594 | 4594 | -134.97 | -105.17 | -6.4419 |
| MurE | [00] 5029 | 5029 | -112.15 | -86.092 | -4.8921 |
| MurE | [01] 9578005 | 9578005 | -133.96 | -111.74 | -5.8026 |
| GalU | [03] 3883 | 3883 | -117.16 | -92.067 | -2.4745 |
| GalU | [03] 4594 | 4594 | -116.76 | -95.276 | -2.9025 |
| **GalU** | **[02] 4679** | **4679** | **-120.4** | **-96.81** | **-2.3821** |
| **GalU** | **[01] 5029** | **5029** | **-118.79** | **-94.081** | **-7.7257** |
| GalU | [02] 9568614 | 9568614 | -107.51 | -88.869 | -3.3719 |
| GalU | [00] 9578005 | 9578005 | -114.08 | -91.543 | -5.2427 |
| NadE | [00] 3883 | 3883 | -139.88 | -115.09 | -5.2771 |
| NadE | [00] 4594 | 4594 | -127.81 | -105.55 | -0.9706 |
| **NadE** | **[00] 4679** | **4679** | **-147.969** | **-120.244** | **-4.95789** |
| **NadE** | **[00] 5029** | **5029** | **-147.25** | **-101.86** | **-1.8108** |
| NadE | [02] 9568614 | 9568614 | -129.25 | -106.97 | -5.2675 |
| **NadE** | **[00] 9578005** | **9578005** | **-149.24** | **-107.48** | **-2.3033** |
| GlmS | [00] 3883 | 3883 | -114.92 | -26.282 | -4.469 |
| GlmS | [00] 4594 | 4594 | -126.57 | -104.58 | -4.8823 |
| **GlmS** | **[00] 4679** | **4679** | **-129.57** | **-104.24** | **-4.9579** |
| **GlmS** | **[03] 5029** | **5029** | **-129.901** | **-102.06** | **-6.7693** |
| GlmS | [00] 9568614 | 9568614 | -124.7 | -102.63 | -5.6877 |
| GlmS | [01] 9578005 | 9578005 | -128.37 | -102.89 | -4.2401 |
| AtpD | [00] 3883 | 3883 | -109.53 | -89.135 | -7.4331 |
| AtpD | [01] 4594 | 4594 | -116.45 | -96.662 | -7.1138 |
| **AtpD** | **[00] 4679** | **4679** | **-133.7** | **-97.178** | **-11.399** |
| AtpD | [01] 5029 | 5029 | -110.21 | -93.545 | -2.1555 |
| AtpD | [00] 9568614 | 9568614 | -118.42 | -99.028 | -7.9538 |
| AtpD | [00] 9578005 | 9578005 | -110.35 | -90.353 | -3.2152 |
| **Def** | **[00] 3883** | **3883** | **-143.93** | **-107.72** | **-4.9117** |
| Def | [00] 4594 | 4594 | -129.93 | -48.342 | -4.9359 |
| Def | [01] 9578005 | 9578005 | -127.91 | -90.407 | -6.2423 |
| Def | [02] 5029 | 5029 | -128.56 | -90.223 | -1.3688 |
| Def | [00] 9568614 | 9568614 | -128.36 | -47.504 | -4.6288 |
| Def | [04]4679 | 4679 | -133.53 | -100.76 | -5.7966 |
| PyrH | [00] 3883 | 3883 | -114.78 | -91.072 | -0.8775 |
| PyrH | [03] 4594 | 4594 | -111.45 | -73.94 | -2.5 |
| **PyrH** | **[01] 4679** | **4679** | **-118.12** | **-85.534** | **-7.8266** |
| **PyrH** | **[03] 5029** | **5029** | **-127.26** | **-92.096** | **-6.4347** |
| PyrH | [02] 9568614 | 9568614 | -106.7 | -55.399 | -1.5237 |
| PyrH | [04] 9578005 | 9578005 | -112.87 | -81.816 | -1.7683 |
